# FXR1 promotes the malignant biological behavior of glioma cells via stabilizing MIR17HG

**DOI:** 10.1186/s13046-018-0991-0

**Published:** 2019-01-28

**Authors:** Shuo Cao, Jian Zheng, Xiaobai Liu, Yunhui Liu, Xuelei Ruan, Jun Ma, Libo Liu, Di Wang, Chunqing Yang, Heng Cai, Zhen Li, Ziyi Feng, Yixue Xue

**Affiliations:** 1 Department of Neurobiology, School of Life Sciences, China Medical University, Shenyang, 110122 People’s Republic of China; 2 Key Laboratory of Cell Biology, Ministry of Public Health of China, China Medical University, Shenyang, 110122 China; 3 Key Laboratory of Medical Cell Biology, Ministry of Education of China, China Medical University, Shenyang, 110122 China; 4 Department of Neurosurgery, Shengjing Hospital of China Medical University, Shenyang, 110004 China; 5 Liaoning Clinical Medical Research Center in Nervous System Disease, Shenyang, 110004 China; 6 Key Laboratory of Neuro-oncology in Liaoning Province, Shenyang, 110004 China; 7 The 102th Class, experimental class of clinical medicine discipline, China Medical University, Shenyang, 110122 Liaoning Province China

**Keywords:** RNA binding proteins, FXR1, Long non-coding RNA, MIR17HG, Glioma cells

## Abstract

**Background:**

Accumulating evidence has highlighted the potential role of RNA binding proteins (RBPs) in the biological behaviors of glioblastoma cells. Herein, the expression and function of RNA binding proteins FXR1 were investigated in human glioma cells.

**Methods:**

Quantitative real-time PCR were conducted to evaluate the expression of MIR17HG and miR-346, miRNA-425-5p in glioma tissues and cells. Western blot were used to explore the expression of FXR1, TAL1 and DEC1 in glioma tissues and cells. Stable knockdown of FXR1 and MIR17HG in glioma cells were established to explore the function of FXR1, MIR17HG in glioma cells. Further, RIP and RNA pull-down assays were used to investigate the correlation between FXR1 and MIR17HG. Cell Counting Kit-8, transwell assays, and flow cytometry were used to investigate the function of FXR1 and MIR17HG in malignant biological behaviors of glioma cells. ChIP assays were employed to ascertain the correlations between TAL1 and MIR17HG.

**Results:**

FXR1and MIR17HG were upregulated in glioma tissues and cell lines. Downregulation of FXR1 or MIR17HG resulted in inhibition of glioma cells progression. We also found that FXR1 regulates the biological behavior of glioma cells via stabilizing MIR17HG. In addition, downregulated MIR17HG increased miR-346/miR-425-5p expression and MIR17HG acted as ceRNA to sponge miR-346/miR-425-5p. TAL1 was a direct target of miR-346/miR-425-5p, and played oncogenic role in glioma cells. More importantly, TAL1 activated MIR17HG promoter and upregulated its expression, forming a feedback loop. Remarkably, FXR1 knockdown combined with inhibition of MIR17HG resulted in the smallest tumor volumes and the longest survivals of nude mice in vivo.

**Conclusions:**

FXR1/MIR17HG/miR-346(miR-425-5p)/TAL1/DEC1 axis plays a novel role in regulating the malignant behavior of glioma cells, which may be a new potential therapeutic strategy for glioma therapy.

**Electronic supplementary material:**

The online version of this article (10.1186/s13046-018-0991-0) contains supplementary material, which is available to authorized users.

## Introduction

Glioblastoma is neuroepithelium-derived and the most malignant form of glioma with poor prognosis [1]. Although the application of advanced surgery, chemotherapy and radiotherapy, patients suffering from glioblastoma meet a median survival of only 12 to 15 months and less than 5% of the patients surviving greater than 5-year [2]. Therefore, revealing the pathogenesis of glioblastoma and finding new effective therapeutic targets have become the focus of current research.

Growing evidence indicates that RNA binding proteins (RBPs) are abnormally expressed in a variety of tumors and are involved in the progression of various human tumors [3–5]. The major biological functions of RBPs include regulation of the RNA stability, splicing, nuclear output and translation [6]. FXR1, located on chromosome 3q26–27, belongs to the Fragile X-Related (FXR) family of RBPs [7]. A recent observation indicated that FXR1 is up-regulated in colorectal cancer tissues and cells, and promoted cell proliferation, migration, and invasion [8]. Moreover, FXR1 is highly expressed in oral squamous cell carcinoma tissues and cells, and inhibits cell senescence by binding and increasing the stability of non-coding RNA TERC [9]. However, far less is known about the role of FXR1-mediated regulation of gene expression in glioma as well as the underlying mechanism.

Long non-coding RNAs (lncRNAs) are defined to be involved in regulating gene expression at transcriptional or post-transcriptional levels, and thus participate in the regulation of cellular physiological and pathological processes [10]. Moreover, accumulated evidence has shown that lncRNAs play an important role in tumorigenesis and progression. As previous reported, LncRNA-UFC1 interacts directly with the mRNA stabilizing protein HuR to increase levels of b-catenin, promotes proliferation and reduces apoptosis in HCC cells [11]. Meanwhile, reduction of MIR155HG inhibited cell proliferation, migration, invasion, and orthotopic glioma growth [12]. RBPs interact with RNA and affect the RNA stability [13]. In cervical carcinoma HeLa cells, RNA binding proteins HuR enhances cell proliferation via binding with and stabilizing OIP5-AS1 [14]. MIR17HG, also known as miR-17-92, is the host gene for the miR-17-92a-1 gene cluster at 13q31 [15]*.* Microarrays from U87 and U251 cells were constructed, and MIR17HG expression was assessed using qPCR. Compared with sh-NC group, MIR17HG expression in sh-FXR1 group was decreased significantly (Additional file 1: Figure S1). However, the expression and potential role of lncRNA MIR17HG in gliomas have not been investigated. Bioinformatics software (Starbase) reveals that FXR1 harbor a putative binding site of MIR17HG, which suggested FXR1 may play a role via increasing the stability of MIR17HG in glioma.

MiRNAs (miRNAs~ 22 nt) are a group of small non-coding RNAs that have been confirmed to be involved in the biological processes of various tumors [16]. In addition, aberrant expressions of miRNAs are ubiquitous in various tumor cells including gliomas, in which miRNAs either act as protooncogenes or tumor suppressor genes [17, 18]. Emerging evidences have confirmed lncRNAs may act as miRNAs sponges to bind to miRNAs and inflect the expression and biological functions of miRNAs [19, 20]. Starbase (http://starbase.sysu.edu.cn/) shows that MIR17HG has putative binding sites with miR-346 and miR-425-5p.

TAL1 (also known as SCL) is a member of the basic helix-loop-helix family of transcription factors and is a critical regulator of hematopoietic and leukemogenesis development [21]. Aberrant expression of TAL1 in later stages of T-cell development is associated with the development of T-cell acute lymphoblastic leukemia (T-ALL) [22]. By binding to the 3’UTR of mRNAs, miRNAs can either suppress the expression of downstream target genes at transcriptional level or degration target mRNA [23, 24]. Using bioinformatic software Targetscan (http://www.targetscan.org/), we predicted TAL1 as a presumed target of miR-346 and miR-425-5p, which indicates that miR-346 and miR-425-5p may be functional in glioma through binding to TAL1. However, the function of TAL1 in glioma remains uncharted.

In the present study, we profiled the expressions of FXR1, MIR17HG, miR-346, miR-425-5p and TAL1 in glioma tissues and cells. We also explored the roles in regulating glioma malignant progression and the interactions among them. This study aims to identify an alternative strategy and targets for the treatment of gliomas.

## Materials and methods

### Human tissue samples

Human glioma specimens and normal brain tissues were obtained from the Department of Neurosurgery at Shengjing Hospital of China Medical University. The study was approved by the Ethics Committee of Shengjing Hospital of China Medical University, and informed consent was obtained from all patients. All specimens were immediately frozen and preserved in liquid nitrogen after surgical resection. According to the WHO classification of tumors in the central nervous system (2007) by neuropathologists. NBTs acquired from fresh autopsy material (donation from individuals who died in a traffic accident and confirmed to be free of any prior pathologically detectable conditions) were used as negative controls.

### Cell culture

Human glioma cell lines (U87, U251) as well as human embryonic kidney (HEK) 293 T cells were purchased from Chinese Academy of Medical Sciences (Beijing, People’s Republic of China). U87 glioma cells and HEK-293 T-cells were cultured in Dulbecco’s modified Eagle medium (DMEM)/high glucose supplemented with 10% fetal bovine serum (FBS, Gibco,Carlsbad, CA, USA), U251 glioma cells were cultured in DMEM/F12 medium supplemented with 10% FBS. All cells were maintained in a humidified incubator at 37 °C with 5% CO_2_.

### RNA extraction and quantitative real-time PCR (qRT-PCR)

Total RNA was extracted from the glioma specimens, normal brain tissues and NHA, U87, and U251 cells using Trizol reagent (Life Technologies Corporation, Carlsbad, CA). The RNA concentration was detected at the 260/280 nm ratio using a Nanodrop Spectrophotometer (ND-100, Thermo, Waltham, MA). The primers of MIR17HG, TAL1, GAPDH, miR-346, miR-425-5p and U6 were synthesized by Thermo Fisher. The primers for each PCR set are listed in Table 1. One-Step SYBR PrimeScript RT-PCR Kit (Takara Bio, Inc., Japan) was used for qRT-PCR detection of MIR17HG, TAL1 and GAPDH. cDNA from miRNAs was synthesized by using TaqMan miRNA Reverse Transcription kit (Applied Biosystems, Foster City, CA, USA). TaqMan Universal Master Mix II was used for TaqMan microRNA assays of miR-346, miR-425-5p and U6 (Applied Biosystems) in the ABI 7500 Fast Real-Time PCR System (Applied Biosystems). Expressions were normalized to endogenous controls and relative quantification (2^−ΔΔCt^) method was used for fold-change calculation.

**Table 1:** Primers used for RT-qPCR

Primer or Probe	Gene	Sequence (5′- > 3′) or Assay ID
Primer	MIR17HG	F: TCAGGAGTTCGAGACCAACC
		R: TGCCTCAGCCTCCAGAGTAG
	TAL1	F: GCCTGGAACGTGAGTGGG
		R: GGATAGTCCCGGGCGTTTTT
Probe	GAPDH miR-346 miR-425-5p U6	F: ACAGTCAGCCGCATCTTCTT R: GCCCAATACGACCAAATCC 4,373,038 4,380,926 4,395,470

### Human lncRNA microarrays

lncRNA analysis, sample preparation, and microarray hybridization were performed by Kangchen Bio-tech (Shanghai, China).

### Cell transfection

Short-hairpin FXR1(sh-FXR1, sequence: 5’-TAAAGTTCGGATGATGAAA-3′), short-hairpin MIR17HG (sh-MIR17HG,sequence:5’-GTGGCCTGCTATTTCCTTCA-3′), short-hairpin TAL1 (sh-TAL1,sequence:5’-GGATGCCTTCCCTATGTTCAC-3′), and DEC1 (sh-DEC1,sequence:5’-GCCCTGCACAGAGAGAGGTCT-3′) plasmids and their respective non-targeting sequence (negative control, NC) (sh-NC); miR-346 agomir (pre-miR-346), miR-346 antagomir (anti-miR-346, sequence: 5’-AGAGGCAGGCAUGCGGGCAGACA-3′), miR-425-5p agomir (pre-miR-425-5p), miR-425-5p antagomir (anti-miR-425-5p, sequence: 5’-UCAACGGGAGUGAUCGUGUCUU-3′) and their respective non-targeting sequence (negative control, NC) (pre-NC or anti-NC) were synthesized (GenePharma, Shanghai, China). TAL1 full length (TAL1 (+)) plasmid and its respective non-targeting sequence (negative control, NC) (TAL1 (+)-NC) were synthesized (GeneScript, Piscataway, NJ, USA). Cells were seeded into 24-well plates and transfected using Lipofectamine 3000 reagent and Opti-MEM I (Life Technologies Corporation, Carlsbad, CA) according to the manufacturer’s instructions. Stable transfected cells were selected through the medium containing Geneticin (G418; Sigma-Aldrich, St Louis, MO, USA), and the transfection efficacy was assessed by qRT-PCR or Western blotting. To evaluate the effect of FXR1 on glioma cells, cells were divided into three groups: control, sh-NC and sh-FXR1 groups. To evaluate the effect of MIR17HG on glioma cells, cells were divided into three groups: control, sh-NC and sh-MIR17HG groups. To determine whether FXR1-mediated regulation of MIR17HG expression could affect the behaviors of glioma cells, cells were divided into five groups: control, sh-FXR1-NC + sh-MIR17HG-NC, sh-FXR1 + sh-MIR17HG-NC, sh-FXR1-NC + sh-MIR17HG and sh-FXR1 + sh-MIR17HG. To evaluate the effect of miR-346 on glioma cells, cells were divided into five groups: control, pre-NC, pre-miR-346, anti-NC and anti-miR-346 groups. To evaluate the effect of miR-425-5p on glioma cells, cells were divided into five groups: control, pre-NC, pre-miR-425-5p, anti-NC and anti-miR-425-5p groups. To determine whether MIR17HG-mediated regulation of miR-346 expression could affect the behaviors of glioma cells, cells were divided into five groups: control, sh-NC + pre-NC, sh-MIR17HG+ pre-miR-346, sh-NC + anti-NC and sh-MIR17HG + anti-miR-346 groups. To determine whether MIR17HG-mediated regulation of miR-425-5p expression could affect the behaviors of glioma cells, cells were divided into five groups: control, sh-NC + pre-NC, sh-MIR17HG+ pre-miR-425-5p, sh-NC + anti-NC and sh-MIR17HG + anti-miR-425-5p groups. To evaluate the effect of TAL1 on glioma cells, cells were divided into five groups: control, TAL1-NC, TAL1, sh-NC and sh-TAL1 groups. To determine whether TAL1 is involved in the miR-346 effect on the behaviors of glioma cells, cells were divided into five groups: control, pre-NC + TAL1-NC, pre-miR-346 + TAL1-NC, pre-NC + TAL1 and pre-miR-346 TAL1 groups. To determine whether TAL1 is involved in the of miR-425-5p effect on the behaviors of glioma cells, cells were divided into five groups: control, pre-NC + TAL1-NC, pre-miR-425-5p + TAL1-NC, pre-NC + TAL1 and pre-miR-425-5p TAL1 groups. To evaluate the effect of DEC1 on glioma cells, cells were divided into three groups: control, sh-NC and sh-DEC1 groups.

### Cell proliferation assay

Cell proliferation was measured by conducting Cell Counting Kit-8 (CCK-8, Dojin, Japan) assay. After transfection, U87 and U251 cells were seeded in 96-well plates at a density of 2000 cells per well. After 72 h, cells per well were added 10 μl CCK-8 solution and incubated for 2 h at 37 °C. The absorbance was recorded at 450 nm with the SpectraMax M5 microplate reader (Molecular Devices, USA).

### Cell migration and invasion assay

U87 and U251 cells migration ability was assessed using 6.5-mm transwell chambers with a pore size of 8 μm (#3422 Costar, Corning, NY, USA). Cells (2 × 10^5^) were suspended in 100 μL serum-free medium and seeded into the upper chamber (or precoated with 80 μL of Matrigel solution (BD, Franklin Lakes, NJ, USA) for cell invasion assay). The lower chamber was filled with 600 μl of 10% FBS medium. After incubation for 48 h, cells that had migrated or invaded to the lower side of the membrane were fixed and stained with 10% Giemsa. Five random fields were chosen to count and take photos under a microscope.

### Quantization of apoptosis by flow cytometry

Cell apoptosis was detected by staining with Annexin V-PE/7AAD (Southern Biotech, Birmingham, AL, USA) according to the manufacturer’s instructions. After washing with phosphate-buffered saline and centrifuging twice, cells were resuspended in Annexin-V binding buffer. They were stained with Annexin V-PE/7AAD and incubated for another 15 min at room temperature in a dark room. Cell samples were analyzed by flow cytometry (FACScan, BD Biosciences) to acquire the apoptotic fractions.

After washing with 4 °C PBS twice, cells were collected and stained with Annexin V-PE/7AAD according to the manufacturer’s instruction. Then the cells were analyzed by flow cytometry (FACScan, BD Biosciences) and apoptotic fractions were investigated by CELL Quest 3.0 software.

### Western blot analysis

Total protein was extracted from cells using RIPA buffer with protease inhibitors (Beyotime Institute of Biotechnology) on ice. Equal amounts of each protein was run on SDS/PAGE gels and electrophoretically transferred to PVDF membranes, which were then incubated in tris-buffered saline containing 5% nonfat milk for 2 h at room temperature followed by incubation with primary antibodies as follows: FXR1 (1:1000, Proteintech, Rosemont, IL), TAL1 (1:500, Santa Cruz, Dallas, TX)), DEC1 (1:1000, Abcam, UK), GAPDH (1:5000, Proteintech, Rosemont, IL). After incubation with secondary antibodies (Goat anti-rabbit or Goat anti-mouse, 1:4000 respectively; Proteintech Group, Chicago), Immunoblots were visualized by electrochemiluminescence detection system and blot bands were scanned using ChemImager 5500 V2.03 software (Alpha Innotech, San Leandro, CA). FLuor Chem2.0 software (AlphaInnotech, San Leandro, CA) was used to calculate the relative integrated density values (IDVs), which were calculated based on GAPDH as an internal control.

### Reporter vector constructs and luciferase reporter assay

The theoretical binding sequence of miR-346(or miR-425-5p)in MIR17HG gene and its mutant sequence were amplified by PCR, synthesized and cloned into a pmirGlo Dual-luciferase vectors (Promega, Madison, WI, USA) to construct dual luciferase reporter vector (GenePharma). HEK293T cells were seeded in 96-well plates and cotransfected with wildtype pmirGLO-MIR17HG (or MIR17HG mutant) reporter plasmid and pre-miR-346 (or pre-miR-425-5p) or pre-NC. Through the Dual-Luciferase Reporter System (Promega), luciferase activity was measured 48 h after transfection. The 3’-UTR sequence of TAL1 containing the putative miR-346(or miR-425-5p)binding sites and their mutant sequences were cloned into Dual-luciferase vectors. The transfection procedure and Luciferase activities measurement were performed similarly as described above.

The MIR17HG and DEC1 promoter regions were amplified from human genomic DNA by PCR. In addition, putative TAL1 binding sites in the PCR conducts were deleted one by one. The PCR products were subcloned into the pGL3-Basic vector (Promega). Human full-length TAL1 gene was constructed in pEX3 vector (GenePharma). Firefly luciferase activity was normalized to renilla luciferase activity for each individual analysis.

### RNA stability measurement

After transfected with sh-NC or sh-FXR1, 2 μg/ml Actinomycin D was added to block de novo RNA synthesis. Total RNA was collected at indicated times and MIR17HG expression was measured by qRT-PCR. The half-life of MIR17HG was determined as the time required to reach 50% of the RNA levels before adding actinomycin D.

### RNA immunoprecipitation (RIP) assay

RIP assays were performed according to the instructions in the Magna RIP RNA-Binding Protein Immunoprecipitation Kit (Millipore, Bedford, MA). Briefly, cell lysate was incubated with RIP buffer containing magnetic beads conjugated with human anti-Ago2 antibody or negative control normal mouse IgG. Samples were incubated with Proteinase K and immunoprecipitated RNA was isolated. Furthermore, RNA was purified from RNA–protein complexes, bound to the beads, and then was analyzed by qRT-PCR.

### RNA pull-down assays

RNA pull-down assay was conducted using Pierce Magnetic RNA-Protein Pull-Down Kit (Thermo fisher) according to the manufacturer’s protocols. Briefly, biotin-labeled MIR17HG or antisense RNA was co-incubated with protein extract of U87 and U251 cells and magnetic beads. The generated bead-RNA-Protein compound was collected by low-speed centrifugation, followed by washing with Handee spin columns. Subsequently, the bead compound was boiled in sodium dodecyl sulfate buffer, and the eluted proteins were detected by Western blotting with GAPDH as the control.

### Chromatin immunoprecipitation (ChIP) assay

ChIP assay was conducted with Simple ChIP Enzymatic Chromatin IP Kit (Cell Signaling Technology, Danvers, Massachusetts, USA) according to the manufacturer’s protocol. Briefly, glioma cells were crosslinked with 1% formaldehyde and collected in lysis buffer. Chromatin was digested with the micrococcal nuclease. 2% aliquots of lysates were used as an input reference and the other immunoprecipitation samples were incubated with normal rabbit IgG or anti-TAL1 antibody at 4 °C with gentle shaking. DNA crosslinks were reversed by 5 mol/l NaCl and proteinase K and then purified. Immunoprecipitated DNA was amplified by PCR using their specific primers. The primers for each PCR set, the sizes of PCR products, and annealing temperatures are listed in Table 2. For each PCR, the corresponding input was taken in parallel for PCR validation.

**Table 2:** Primers used for ChIP experiments

Gene	Binding site or Control	Sequence (5′- > 3′)	Product size (bp)	Annealing temperature (°C)
MIR17HG	PCR1	F: TGTTTATGAGGTTCTTTCCGAGC	160	59.2
		R: GTGTTTCCTTAACCTGGCCG		
	PCR2	F: TGCTTGTATGTCAGATTTCCACA	168	58.6
		R: TCACACTCTCTGAATCATGGGA		
	PCR3	F:CTGAGGGCTATATAGAACTGCTCTGACG	140	56
		R:TGAAATGCCAAAGTGAGACACACAGC		
DEC1	PCR1	F: CTTGACCAAAACTCCACAGTGA	102	58.2
		R:AGTTGCAAATGTTACCAAATTTACAA		
	PCR2	F: TGCTTCTCTGAATGAGGTGAACT	138	59.8
		R: AGGTTTCTCAGTGCTTACAACCA		
	PCR3	F: GCTAACAAAACCCAATTTCTTCTGG	186	59.6
		R: TCCTCAGCAGACATATGGCC		
	PCR4	F:CATTCTATCCTCTGATCCCTGGAACC	184	56
		R: CACTTACTCTGCGCCACGTTCTG		

### Nascent RNA capture assay

Nascent RNA capture assay were performed using the Click-iT Nascent RNA Capture Kit (Invitrogen) following the manufacturer’s protocol. Briefly, cells were incubated in 5-ethymyl uridine (EU) and total RNA labeled with EU was isolated using TRIzol reagent (Invitrogen). Subsequently, EU-labeled RNA was biotinylated in a Click-iT reaction buffer and then captured using streptavidin magnetic beads.

### Tumor xenografts in nude mice

All animal procedures were performed in accordance with the Care and Use of Laboratory Animals and protocols approved by the Animal Care Committee of Shengjing Hospital. Four weeks old female BALB/C athymic nude mice were obtained from the National Laboratory Animal Center (Beijing, People’s Republic of China). Animals were in line with the guidelines of the laboratory animal center. For the in vivo study, the stably transfected cells with sh-FXR1-NC + sh-MIR17HG-NC, sh-FXR1 + sh-MIR17HG-NC, sh-FXR1-NC + sh-MIR17HG, sh-FXR1 + sh-MIR17HG were picked as described above. A suspension of 3 × 10^5^ cells in a 100 μl volume was subcutaneously injected into the right flanks of mice. The tumor volume was evaluated every 4 days and calculated by the formula: volume (mm^3^) = length × width^2^/2. The mice were sacrificed and tumors were isolated on the 40th day postinoculation. As for intracranial orthotopic inoculation, 3 × 10^5^ cells were implanted into the right striatum of mice stereotactically. The number of survived nude mice was registered, and survival analysis was determined using Kaplan–Meier survival curve.

### Statistical analysis

Data are presented as mean ± SD from at least three independent experiments. All statistical analyses were performed by SPSS 18.0 statistical software (IBM, New York, NY) with the Student’s t-test (two tailed) or one-way analysis of variance for multiple groups. Survival analysis was evaluated using the Kaplan-Meier method and assessed using the log-rank test. Differences were considered statistically significant when *P* < 0.05.

## Results

### FXR1 was upregulated in glioma tissues and cell lines, knockdown of FXR1 inhibited the malignant biological behavior of glioma cells

**Fig. 1:**
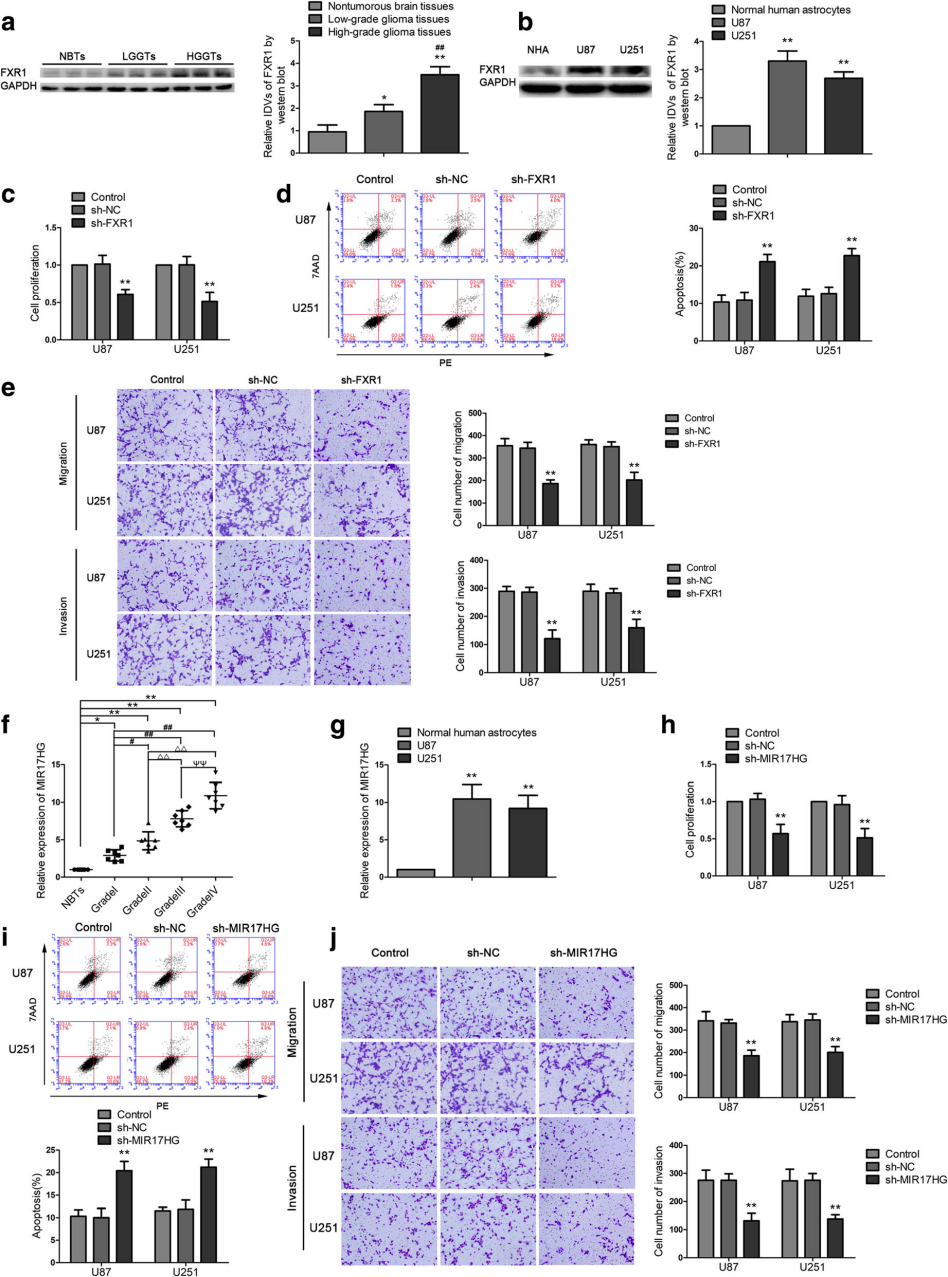
The expression and effect of FXR1 and MIR17HG on the biological behavior of glioma cells. **a** FXR1 protein expression levels in nontumorous brain tissues (NBTs), low-grade glioma tissues (WHO I-II), and high-grade glioma tissues (WHO III-IV). Data are presented as the mean ± SD (*n* = 3 in each group). *P < 0.05, **P < 0.01 versus NBTs group; ^##^*P* < 0.01 versus low-grade glioma tissues group. **b** FXR1 protein expression levels in normal human astrocytes (NHA), U87 and U251 cells. Data are presented as the mean ± SD (*n* = 3 in each group). **P < 0.01 versus NHA group. **c**, **h** CCK-8 assay was conducted to investigate the effect of FXR1 (MIR17HG) on proliferation of U87 and U251 cells. **d**, **i** Flow cytometry analysis to evaluate the effect of FXR1 (MIR17HG) on the apoptosis of U87 and U251 cells. **e**, **j** Transwell assays were used to measure the effect of FXR1 (MIR17HG) on cell migration and invasion of U87 and U251 cells. Data are presented as the mean ± SD (*n* = 3 in each group). *P < 0.05, **P < 0.01 versus sh-NC group (empty vector); Scale bars represent 40 μm. **f** The expression of MIR17HG in glioma tissues of different grades and NBTs. Data are presented as the mean ± SD (*n* = 7 in each group). *P < 0.05, **P < 0.01 versus NBTs group; ^#^*P* < 0.05, ^##^*P* < 0.01 versus Grade I group; ^△△^*P* < 0.01 versus Grade II group; ^ΨΨ^*P* < 0.01 versus Grade III group. **g** The expression of MIR17HG in NHA and glioma cell lines (U87 and U251). Data are presented as the mean ± SD (*n* = 3 in each group); **P < 0.01 versus NHA group. Using one-way analysis of variance for statistical analysis

The expression of FXR1 in glioma tissues and cells were detected by western blot. As shown in Fig. 1a, FXR1 expression was significantly increased in glioma tissues of different grades compared with normal brain tissues (NBTs), and the expression was positively correlated with the pathological grade of glioma tissues. Moreover, FXR1 expression was significantly up-regulated in U87 and U251 cell lines compared with the normal human astrocytes (NHAs) group (Fig. 1b). Stable FXR1 silenced constructs were used to assess the functional role of FXR1 in U87 and U251 cells (transfection efficiency of sh-FXR1 data was shown in Additional file 2: Figure S2a). As shown in Fig. 1c, the Cell Counting Kit-8 (CCK8) assay revealed that proliferation rate of U87 and U251 cells was decreased in the sh-FXR1 group compared with the sh-NC group. Transwell assays showed that the numbers of migrated and invaded cells in the sh-FXR1 group were significantly decreased compared with the sh-NC group (Fig. 1e). Flow cytometry analysis demonstrated that silence of FXR1 remarkedly increased apoptosis of glioma cells (Fig. 1d).

### FXR1 regulates the biological behavior of glioma cells via stabilizing MIR17HG

**Fig. 2:**
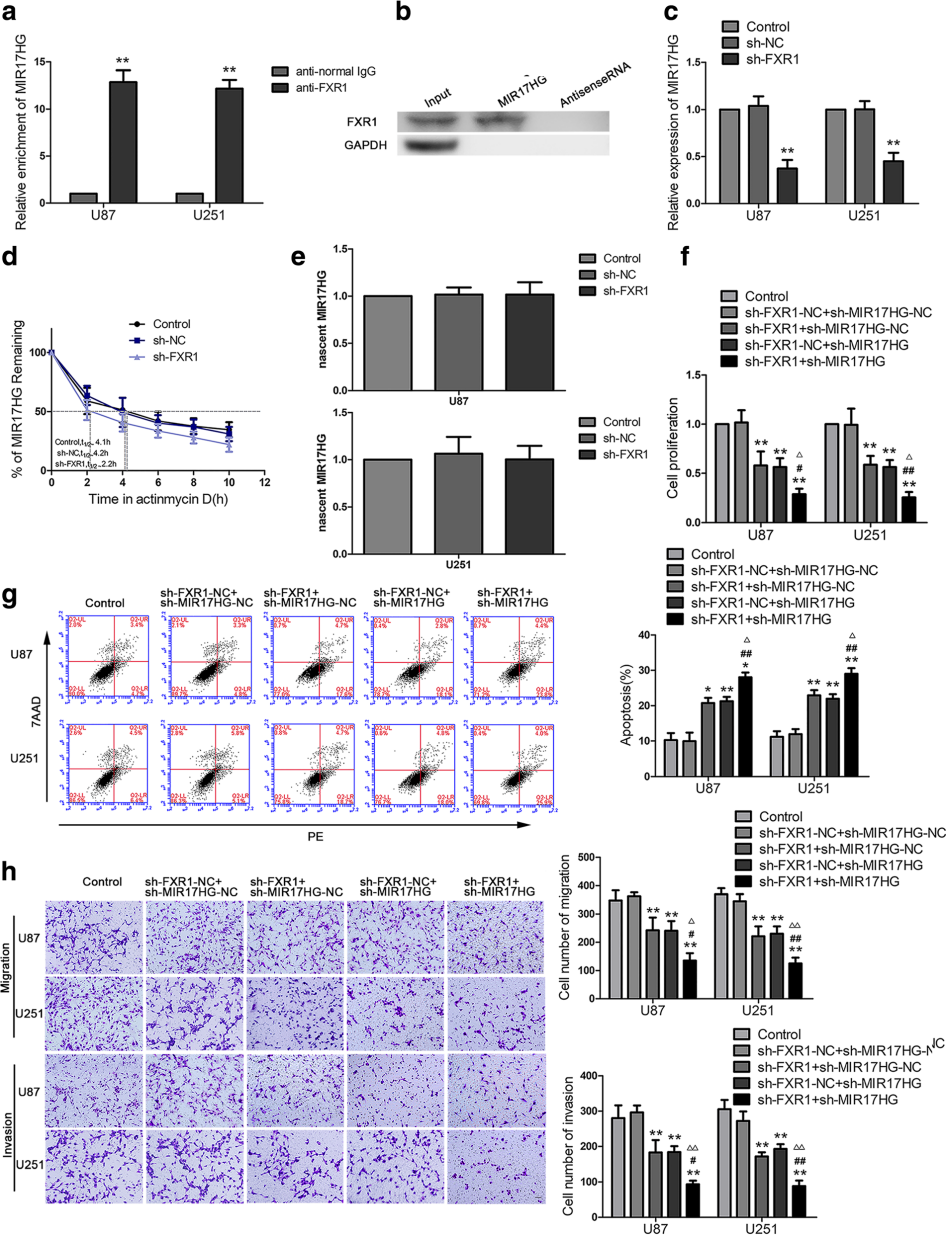
FXR1 bound with MIR17HG and promotes the effect of MIR17HG on glioma. **a** MIR17HG was identified in the FXR1 complex. Relative enrichment of MIR17HG was measured using real-time qPCR. Data represent mean ± SD (n = 3 in each group). **P < 0.01 versus anti-normal IgG group, using Student’s t test. **b** FXR1 and GAPDH protein levels in immunoprecipitation with MIR17HG RNA were evaluated by western blots. The expression levels of FXR1 and GAPDH proteins are shown. **c** Real-time qPCR analysis for FXR1 regulating MIR17HG expression in U87 and U251 cells. Data are presented as the mean ± SD (*n* = 3 in each group). **P < 0.01 versus sh-NC group. **d** The graph represents the relative levels of the MIR17HG at the different actinomycin D treatment times in the control group, sh-NC group, and sh-FXR1 group. **e** Click-iT Nascent RNA capture kit (Life Technology) was conducted to label and capture newly synthesized RNA, and nascent MIR17HG was measured using real-time qPCR. **f** CCK-8 assay was conducted to investigate the effect of FXR1 and MIR17HG inhibition on proliferation in U87 and U251 cells. **g** Flow cytometry analysis of U87 and U251 cells treated with inhibition of FXR1 and MIR17HG. **h** Quantification number of migration and invasion cells treated with inhibition of FXR1 and MIR17HG. Representative images and accompanying statistical plots were presented. Data are presented as the mean ± SD (*n* = 3 in each group). *P < 0.05, **P < 0.01 versus sh-FXR1-NC + sh-MIR17HG-NC group (empty vector); ^#^*P* < 0.05, ^##^*P* < 0.01 versus sh-FXR + sh-MIR17HG-NC group; ^△^*P* < 0.05, ^△△^*P* < 0.01 versus sh-FXR1-NC + sh-MIR17HG group. Scale bars represent 40 μm. Using one-way analysis of variance for statistical analysis

Using lncRNAs microarray, we found MIR17HG was significantly downregulated in glioma cells treated with sh-FXR1 (Additional file 1: Figure S1). Therefore, we predicted that MIR17HG was involved in FXR1-mediated regulation on glioma cells. Further, the expressions of MIR17HG in glioma tissues and cells were investigated by qPCR. As shown in Fig. 1f, MIR17HG expression was increased and positively correlated with the progression of glioma pathological grades. MIR17HG was significantly upregulated in glioma cell lines compared with normal human astrocytes (Fig. 1g). Glioma cells stably expressing sh-MIR17HG were established to investigate the function of MIR17HG. The transfection efficiency of MIR17HG was confirmed (Additional fie 2: Figure S2b). In functional experiments, sh-MIR17HG impeded cell proliferation (Fig. 1h), hindered cell migration and invasion (Fig. 1j), and meanwhile, promoted apoptosis compared to sh-NC group (Fig. 1i). Further, we investigated the correlation between FXR1 and MIR17HG. We predicted FXR1 might bind to MIR17HG with the help of bioinformatics software (Starbase). RNA immunoprecipitation (RIP) results showed that enrichment of MIR17HG in the anti-FXR1 group was significantly higher than that in the anti-normal IgG group (Fig. 2a). Also, RNA pull-down assays demonstrated that MIR17HG bound with FXR1 (Fig. 2b). We further measured MIR17HG expression in the sh-FXR1 group by qPCR. As shown in Fig. 2c, MIR17HG expression was significantly decreased in the sh-FXR1 group compared with the sh-NC group. As shown in Fig. 2d, the half-life of MIR17HG was significantly decreased in sh-FXR1 cells treated with actinomycin D. Moreover, novel MIR17HG was analyzed using Click-iT Nascent RNA Capture to label and isolate newly synthesized RNA. qPCR demonstrated that novel MIR17HG level in sh-NC group and sh-FXR1 group is not statistically significant (Fig. 2e). In addition, inhibition of FXR1 combined with inhibition of MIR17HG significantly impeded glioma cell growth. As shown in Fig. 2f-h, compared with sh-FXR1-NC + sh-MIR17HG-NC group, the proliferation, migration and invasion abilities of U87 and U251 cells were decreased in thesh-FXR1 + sh-MIR17HG-NC, sh-FXR1-NC + sh-MIR17HG and sh-FXR1 + sh-MIR17HG groups, and the apoptosis of glioma cells were significantly increased. Moreover, the glioma cells treated with sh-FXR1 and sh-MIR17HG exhibited weaker proliferation, migration and invasion abilities, and higher apoptosis ratio. These results indicated that FXR1 facilitated glioma cells malignant progression by stabilizing MIR17HG.

### MIR17HG targets miR-346 and miR425-5p, and the expressions of miR-346 and miR-425-5p are negatively correlated with MIR17HG

**Fig. 3:**
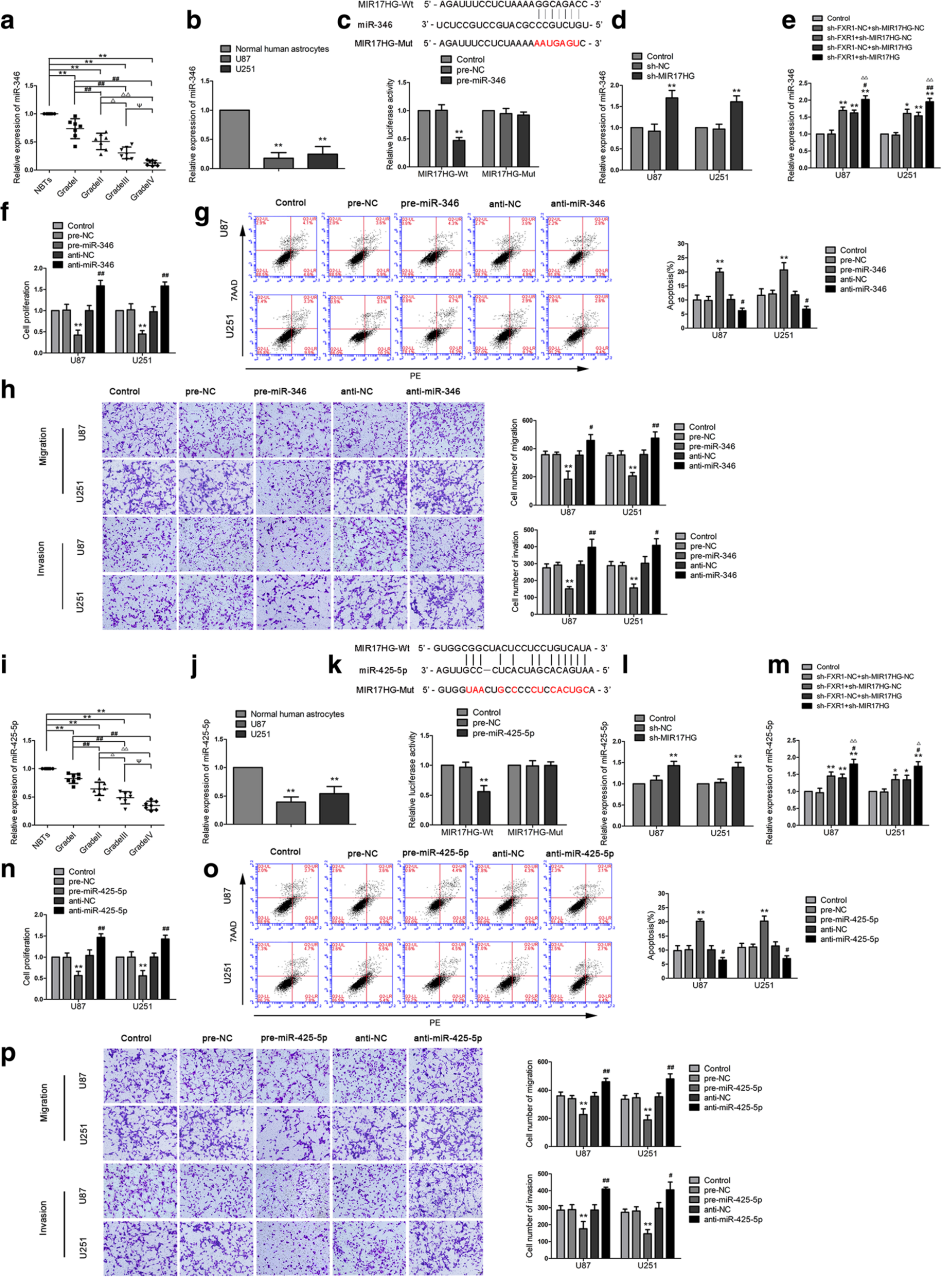
Overexpression of miR-346 and miR-425-5p inhibited the malignant progression of glioma cells. **a**, **i** Expression levels of miR-346 (miR-425-5p) in NBTs and glioma tissues. Data are presented as the mean ± SD (*n* = 7 in each group). **P < 0.01 versus NBTs group; ^##^*P* < 0.01 versus Grade I group; ^△^*P* < 0.05, ^△△^*P* < 0.01 versus Grade II group; ^Ψ^*P* < 0.05, ^ΨΨ^*P* < 0.01 versus Grade III group. **b**, **j** Expression levels of miR-346 (miR-425-5p) in NHA and glioma cell lines. Data are presented as the mean ± SD (n = 3 in each group). **P < 0.01 versus NHA group. **c**, **k** The predicted miR-346 (miR-425-5p) binding sites in the MIR17HG (MIR17HG-Wt) and/or the designed mutant sequence (MIR17HG-Mut) are indicated. Relative luciferase activity was conducted after cells were transfected with MIR17HG-Wt or MIR17HG-Mut. **P < 0.01 versus MIR17HG-Wt + pre-NC. **d**, **l** Real-time qPCR analysis for MIR17HG regulating miR-346 (miR-425-5p) expression in U87 and U251 cells. **P < 0.01 versus sh-NC group. **e**, **m** Real-time qPCR analysis for FXR1 and MIR17HG regulating miR-346 (miR-425-5p) expression in U87 and U251 cells. Data were presented as the mean ± SD (*n* = 3 in each group). *P < 0.05, **P < 0.01 versus sh-FXR1-NC + sh-MIR17HG-NC group; ^#^*P* < 0.05, ^##^*P* < 0.01 versus sh-FXR + sh-MIR17HG-NC group; ^△^*P* < 0.05, ^△△^*P* < 0.01 versus sh-FXR1-NC + sh-MIR17HG group. **f**, **n** CCK-8 assay was used to measure the proliferative effect of miR-346 (miR-425-5p) on U87 and U251 cells. **g**, **o** Flow cytometry analysis of U87 and U251 with the altered expression of miR-346 (miR-425-5p). **h**, **p** Transwell assays were used to measure the effect of miR-346 (miR-425-5p) on cell migration and invasion of U87 and U251 cells. Data are presented as the mean ± SD (*n* = 3 in each group). **P < 0.01 versus pre-NC group; ^#^*P* < 0.05, ^##^*P* < 0.01 versus anti-NC group. Scale bars represent 40 μm. Using one-way analysis of variance for statistical analysis

In order to further study the molecular mechanism of FXR1 in glioma cells via stabilizing MIR17HG, we studied the interaction between MIR17HG and downstream miRNAs. According to the bioinformatics database (Starbase), we proposed that MIR17HG might harbor miR-346 and miR-425-5p binding sites. miR-346 and miR-425-5p expression levels in glioma tissues and cells were analyzed by qPCR. As shown in Fig. 3a and i, miR-346 and miR-425-5p expression level were negatively correlated with the pathological grades of glioma. Meanwhile, the expressions of miR-346 and miR-425-5p were lower in U87 and U251 glioma cells than that in NHAs (Fig. 3b, j). To investigate the miR-346/miR-425-5p effect on glioma cells, pre-miR-346/pre-miR-425-5pand anti-miR-346/anti-miR-425-5p were transfected into U87 and U251 cells, respectively. After transfection, we first examined the transfection efficiency (Additional file 2: Figure S2c). In functional experiments, overexpressed miR-346/miR-425-5p inhibited cell proliferation (Fig. 3f, n), decreased migrating and invading cell numbers (Fig. 3g, o) and significantly induced apoptosis compared with the pre-NC group (Fig. 3h, p). Meanwhile, downregulation of miR-346/miR-425-5p has the opposite effect.

**Fig. 4:**
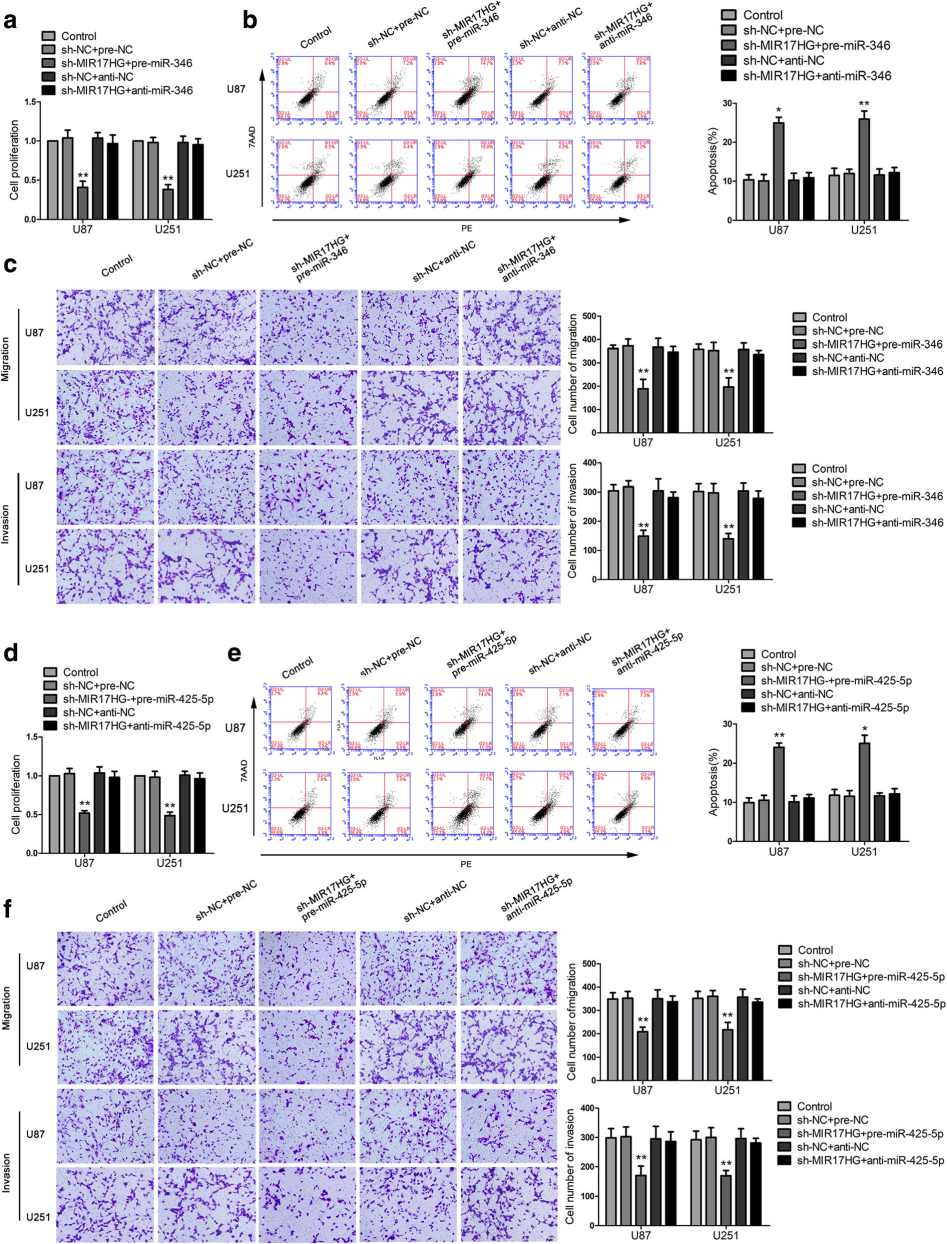
Knockdown of MIR17HG impaired malignant biological behaviors of glioma cells by inducing miR-346 (miR-425-5p) expression. **a**, **d** CCK-8 assay was used to determine the proliferative effect of MIR17HG and miR-346 (miR-425-5p) on U87 and U251 cells. **b**, **e** Flow cytometry analysis of U87 and U251 with the altered expression of MIR17HG and miR-346 (miR-425-5p). **c**, **f** Quantification number of migration and invasion cells with the altered expression of MIR17HG and miR-346 (miR-425-5p). Representative images and accompanying statistical plots were presented. Data are presented as the mean ± SD (*n* = 3 in each group). *P < 0.05, **P < 0.01 versus sh-NC + pre-NC group (empty vectors). Scale bars represent 40 μm. Using one-way analysis of variance for statistical analysis

To verify our prediction, dual-luciferase gene reporter assays were conducted to assess the binding site of MIR17HG and miR-346/miR-425-5p. The luciferase activity in the MIR17HG-Wt + pre-miR-346group/MIR17HG-Wt + pre-miR-425-5p group was dramatic decline than that in the pre-NC group, whereas the luciferase activity in the MIR17HG-Mut group was not affected (Fig. 3c, k). Further, we detected the expression of miR-346 and miR-425-5p in sh-MIR17HG cells by qPCR. The expressions of miR-346 and miR-425-5p were increased in the sh-MIR17HGgroup compared with the sh-NC group (Fig. 3d, l). Also, expression of miR-346 and miR-425-5p were markedly upregulated in cells treated with sh-FXR1 + sh-MIR17HG (Fig. 3e, m). To clarify whether miR-346andmiR-425-5p were involved inMIR17HG-mediatedregulation on glioma cells, miR-346/miR-425-5p overexpression and knockdown were transfected to stable sh-MIR17HG cells. The proliferation, migration, invasion abilities and apoptosis ratio of glioma cells in the sh-NC + pre-NC group, sh-NC + anti-NC group and sh-MIR17HG + anti-miR-346 group were not significantly different from those in the control group. Compared with the sh-NC + pre-NC group, the cell proliferation, migration and invasion abilities of sh-MIR17HG + pre-miR-346 group were decreased significantly, and the apoptosis ratio was markedly enhanced (Fig. 4a-c). Similar results were observed in cells treated with miR-425-5p (Fig. 4d-f).

**Fig. 5:**
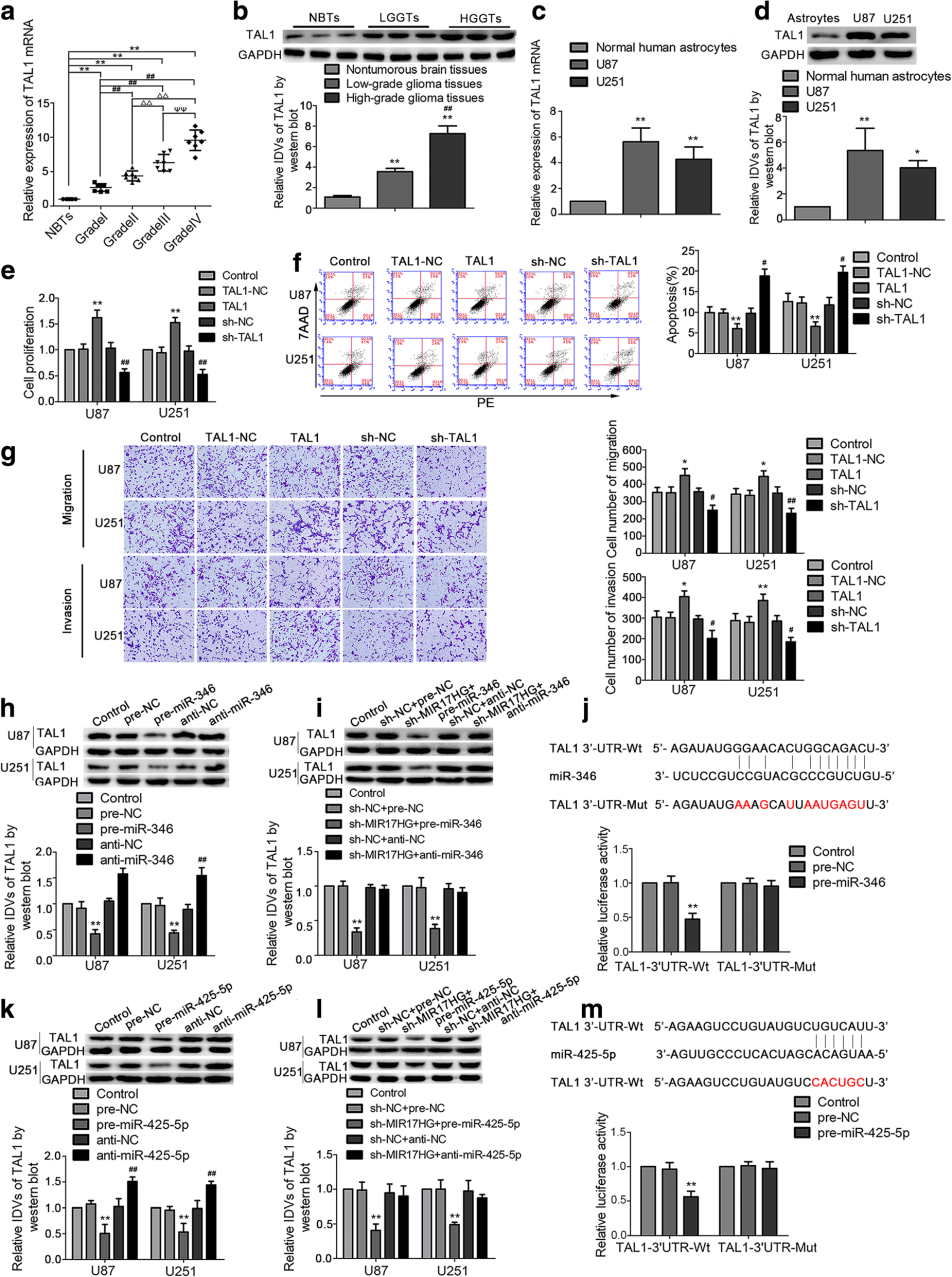
TAL1 was upregulated in glioma tissues and cells and exerted oncogenic function in glioma cells. **a** TAL1 mRNA expression levels in NBTs and glioma tissues. Data are presented as the mean ± SD (n = 7 in each group). **P < 0.01 versus NBTs group; ^##^*P* < 0.01 versus Grade I group; ^△△^*P* < 0.01 versus Grade II group; ^ΨΨ^*P* < 0.01 versus Grade III group. **b** TAL1 protein expression levels in NBTs and glioma tissues. **P < 0.01 versus NBTs group; ^*##*^*P* < 0.01 versus low-grade glioma tissues group. **c**, **d** The expression levels of TAL1 mRNA and protein in NHA, U87 and U251 cells. Data are presented as the mean ± SD (*n* = 3 in each group); *P < 0.05, **P < 0.01 versus NHA group. **e** CCK-8 assay was used to explore the effect of TAL1 on proliferation in U87 and U251 cells. **f** Flow cytometry analysis of U87 and U251 with different expression of TAL1. **g** Transwell assays were used to measure the effect of TAL1 on cell migration and invasion of U87 and U251 cells. *P < 0.05, **P < 0.01 versus TAL1-NC group; ^#^*P* < 0.05, ^##^*P* < 0.01 versus sh-NC group. Scale bars represent 40 μm. **h**, **k** Western blot assay were used to detect the TAL1 expression after miR-346 (miR-425-5p) over-expression or knockdown. **P < 0.01 versus pre-NC group; ^##^*p* < 0.01 versus anti-NC group. **i**, **l** Western blot assay were used to detect the TAL1 expression regulated by MIR17HG and miR-346 (miR-425-5p). **P < 0.01 versus sh-NC + pre-NC group. **j**, **m** The predicted miR-346 (miR-425-5p) binding sites in the 3’UTR region of TAL1 (TAL1–3’UTR-Wt) and the designed mutant sequence (TAL1–3’UTR-Mut) are indicated. Relative luciferase activity was conducted after cells were transfected with TAL1–3’UTR-Wt or TAL1–3’UTR-Mut. Data are presented as the mean ± SD (n = 3 in each group). **P < 0.01 versus TAL1-Wt + pre-NC group. Using one-way analysis of variance for statistical analysis

Using bioinformatics databases (TargetScan), TAL1 was identified as a putative downstream gene of miR-346 and miR-425-5p. We first detected the expression of TAL1 in glioma tissues and cells. As shown in Fig. 5a and b, TAL1mRNA and protein expression was upregulated in glioma tissues compared with that in NBTs. Likewise, TAL1 mRNA and protein expression was robustly upregulated in glioma U87 and U251 cells (Fig. 5c, d). Further, we investigated the effect of TAL1 on glioma cells. After transfection, we first examined the transfection efficiency (Additional file 2: Figure S2d). Glioma cells treated with TAL1 inhibition had decreased proliferation, migrating and invading glioma cell numbers and increased apoptosis ratio compared with that in sh-NC group, whereas overexpressed TAL1 induced enhanced cell proliferation (Fig. 5e), promoted cell migration and invasion ability (Fig. 5f) and significantly inhibited cell apoptosis (Fig. 5g). These results suggested TAL1 promoted malignant progression of glioma cells.

To support our hypothesis that TAL1 was a direct target of miR-346 and miR-425-5p, conducted dual-luciferase gene reporter assays were performed to clarify the interaction between TAL1 and miR-346/miR-425-5p. As shown in Fig. 5j and m, luciferase activity in the TAL1-wt + pre-miR-346/TAL1-wt + pre-miR-425-5p group was significantly attenuated than that in pre-NC group, while TAL1-Mut groups were not affected. Further, we detected the TAL1 mRNA and protein expression in cells treated with pre-miR-346/pre-miR-425-5p or anti-miR-195/anti-miR-425-5p. As expected, TAL1 mRNA and protein expression were diminished in the pre-miR-346/pre-miR-425-5p group than that in pre-NC group, whereas the expression of TAL1 in anti-miR-346 /anti-miR-425-5p group showed the contrary result (Fig. 5h, k, Additional file 3: Figure S3a, c). Also, TAL1 mRNA and protein expression were diminished in the sh-MIR17HG + pre-miR-346/sh-MIR17HG + pre-miR-425-5p group than that in the sh-NC + pre-NC group (Fig. 5i, l, Additional file 3: Figure S3b, d).

### TAL1 mediated the tumor-suppressive effects of miR-346 and miR-425-5p in glioma cell lines

**Fig. 6:**
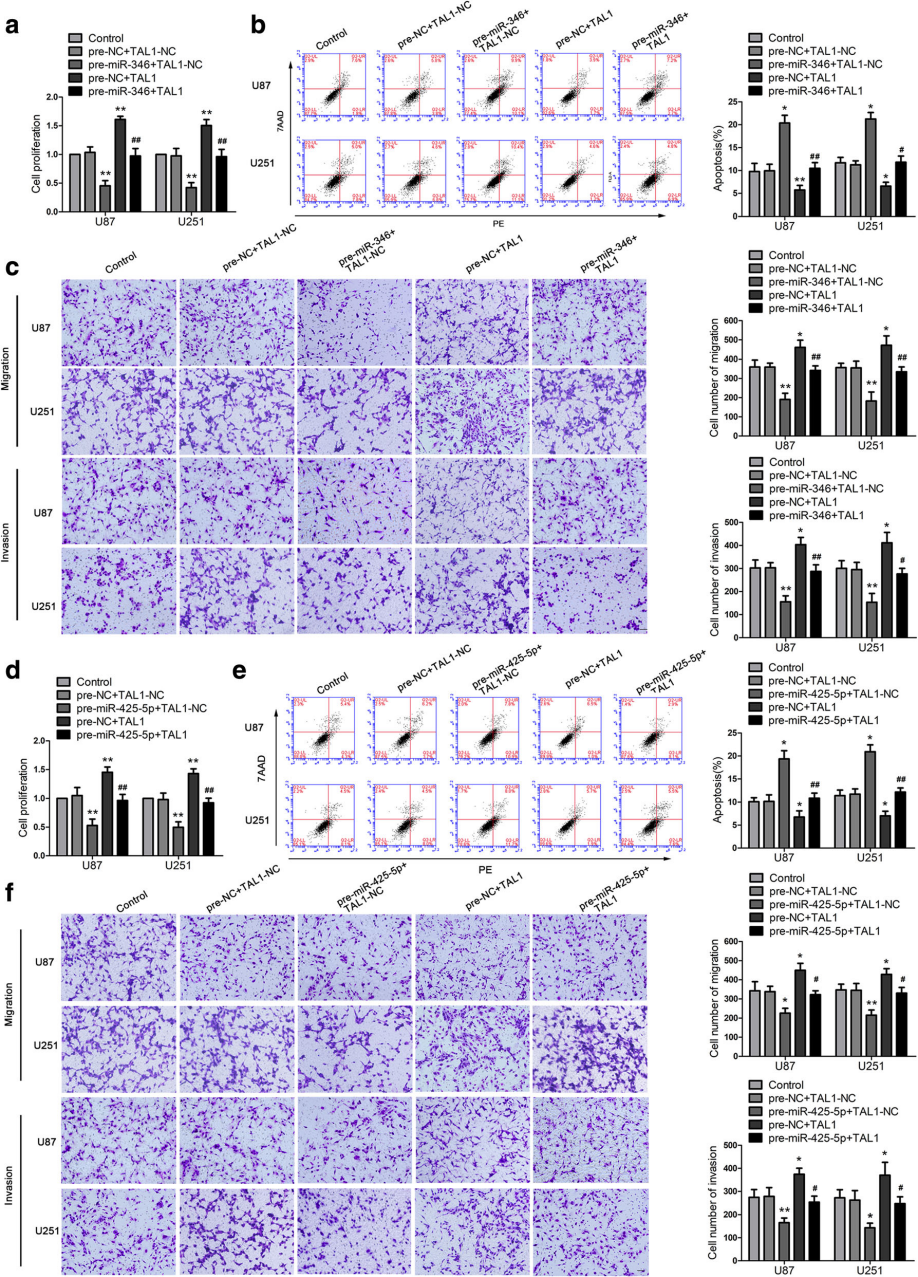
TAL1 was involved in miR-346 (miR-425-5p)-mediated tumor-suppressive function in glioma cells. **a** and **d** CCK-8 assays were performed on U87 and U251 cells with the altered expression of miR-346 (miR-425-5p) and TAL1. **b** and **e** Flow cytometry analysis of U87 and U251 cells with the altered expression of miR-346 (miR-425-5p) and TAL1. **c** and **f** Quantification number of migration and invasion cells with the altered expression of miR-346 (miR-425-5p) and TAL1. Representative images and accompanying statistical plots were presented. Data are presented as the mean ± SD (n = 3 in each group). *P < 0.05, **P < 0.01 versus pre-NC + TAL1-NC group (empty vector); ^#^*P* < 0.05, ^##^*P* < 0.01 versus pre-miR-346 (pre-425-5p) + TAL1-NC group. Scale bars represent 40 μm. Using one-way analysis of variance for statistical analysis

**Fig. 7:**
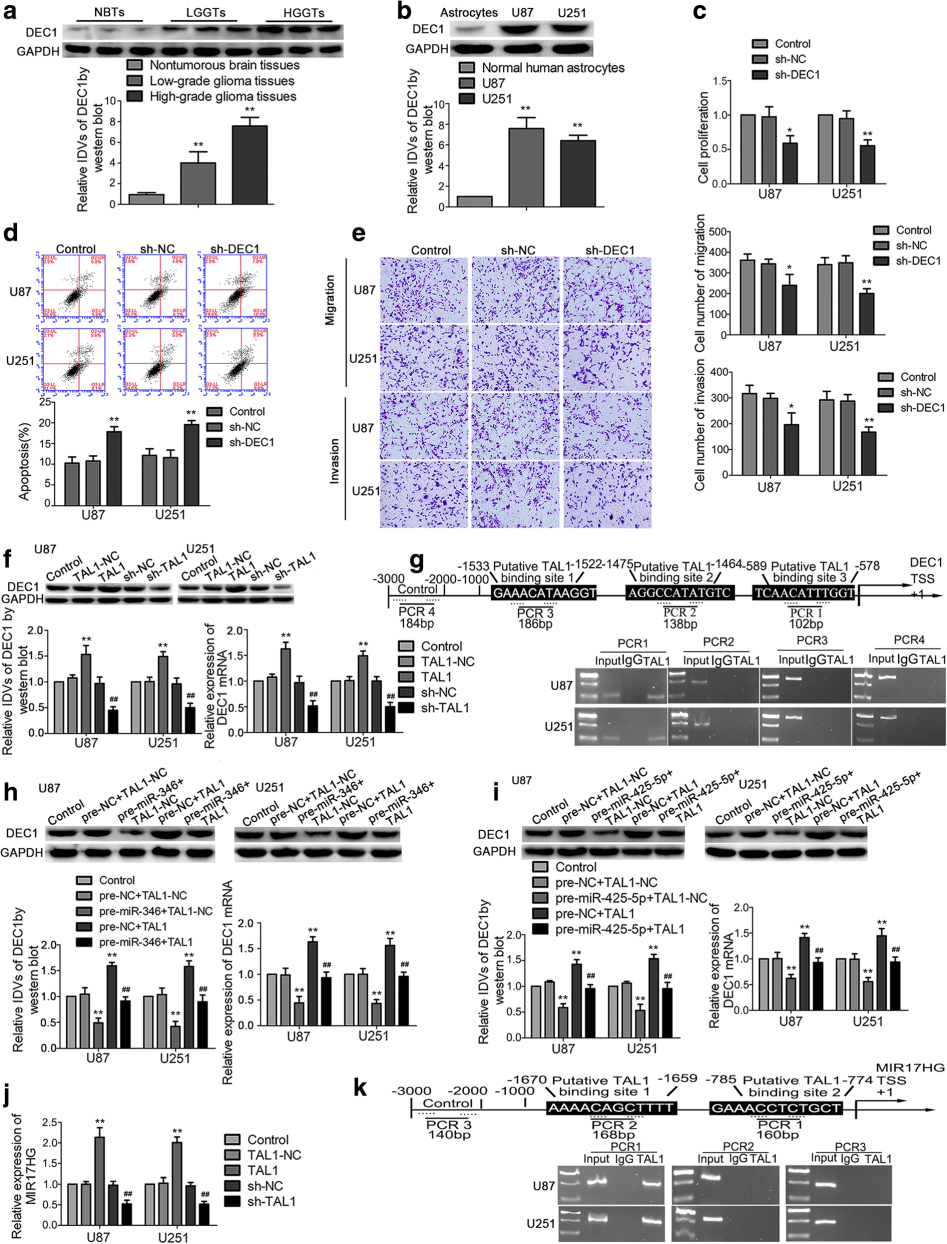
DEC1 was upregulated in glioma tissues and cells and played oncogenic role in glioma cells. **a** DEC1 protein expression levels in NBTs and glioma tissues. **P < 0.01 versus NBTs group; ^##^*P* < 0.01 versus low-grade glioma tissues group. **b** DEC1 protein expression levels in astrocytes, U87, and U251 cells. Data are presented as the mean ± SD (n = 3 in each group). **P < 0.01 versus human normal astrocytes group. **c** CCK-8 assay was used to measure the proliferation effect of DEC1 on U87 and U251 cells. **d** Flow cytometry analysis of U87 and U251 cells with the altered expression of DEC1. **e** Transwell assays were used to measure the effect of DEC1 on cell migration and invasion of U87 and U251 cells. Scale bars represent 40 μm. Data are presented as the mean ± SD (n = 3 in each group). *P < 0.05, **P < 0.01 versus sh-NC group. **f** qRT-PCR and western blot analysis for TAL1 regulating DEC1 expression in U87 and U251 cells. **P < 0.01 versus TAL1-NC group; ^##^*P* < 0.01 versus sh-NC group. **g** TAL1 bound to the promoter of DEC1 in U87 and U251 glioma cells. Putative TAL1 binding sites are indicated. Immunoprecipitated DNA was amplified by PCR. Normal rabbit IgG was used as a negative control. **h**, **i** qRT-PCR and western blot assay were used to detect the DEC1 expression regulated by miR-346 (miR-425-5p) and TAL1. **P < 0.01 versus pre-NC + TAL1-NC group; ^##^*P* < 0.01 versus pre-miR-346 (pre-miR-425-5p) + TAL1-NC group. **j** Real-time qPCR analysis for TAL1 regulating MIR17HG expression in U87 and U251 cells. Data are presented as the mean ± SD (n = 3 in each group). **P < 0.01 versus TAL1-NC group; ^#^*P* < 0.05 versus sh-NC group. **k** TAL1 bound to the promoter of MIR17HG in U87 and U251 glioma cells. Putative TAL1 binding sites are indicated. Immunoprecipitated DNA was amplified by PCR. Normal rabbit IgG was used as a negative control. Using one-way analysis of variance for statistical analysis

To explore whether TAL1 mediated the tumor-suppressive effects ofmiR-346 and miR-425-5p in glioma cell lines, stable TAL1-silenced glioma cells were transfected with pre-miR-346/pre-miR-425-5p.Compared with pre-NC + TAL1-NC group, the proliferation, migration and invasion abilities of glioma cells were reduced in the pre-miR-346/pre-miR-425-5p + TAL1-NC group, while increased in the pre-NC + TAL1 group. Cell apoptosis ratio was increased in the pre-miR-346/pre-miR-425-5p + TAL1-NC group, and was decreased in the pre-NC + TAL1 group. In addition, in the pre-miR-346 + TAL1 group, TAL1 rescued the inhibitory effect of pre-miR-346 + TAL1-NC on the proliferation, migration and invasion of glioma cells (Fig. 6a–c). Overexpression of TAL1 hindered the increase in the apoptosis ratio caused by pre-miR-346+ TAL1-NC group. Moreover, miR-425-5p was also detected to show the similar effects (Fig. 6d-f). Western blot was used to clarify whether miR-346/miR-425-5p inhibited DEC1 expression by down-regulating TAL1. Compared with the pre-NC + TAL1-NC group, DEC1 expression was decreased in the pre-miR-346/pre-miR-425-5p + TAL1-NC group, while overexpression of TAL1 rescued the effect (Fig. 7h, i).

### Oncogene DEC1 is involved in TAL1-mediated regulation of glioma cell malignant progression

DEC1 was identified as a direct downstream target of TAL1 by bioinformatic databases (JASPAR). Firstly, DEC1 expression in human glioma tissues and cells were detected. Compared to NBTs, DEC1 expression was significantly upregulated in glioma tissues and positively correlated with the pathological grades (Fig. 7a). The expression level of DEC1 in U87 and U251 glioma cells were higher than that in NHA cells (Fig. 7b). We further explore the effect of the DEC1 on glioma cells, the transfection efficiency of DEC1 was determined by western blot (Additional file 2: Figure S2e). As shown in Fig. 7c-e, cell proliferation, migration, and invasion abilities were impeded in the sh-DEC1 group compared with those in sh-NC group, while the apoptosis was increased. To clarify whether DEC1 was involved in the tumor-promotive role of TAL1, the expression levels of DEC1 in transfected glioma cells were also assessed. As shown in Fig. 7f, DEC1 mRNA and protein expression were increased in the TAL1 group, but decreased in the sh-TAL1 group compared with the NC group. In the rescue experiment, DEC1 expression levels that were supposed to be decreased by the overexpression of miR-346/miR-425-5p were elevated by the overexpression of TAL1 (Fig. 7h, i).

To determine whether TAL1 might be binding to the DEC1 promoter, luciferase assays were conducted. The putative TAL1 binding sites in the promoter reporter constructs were deleted one by one. As shown in Additional file 4: Figure S4a, deletion of the -1533 and -1475 site did not induce a significant change in DEC1 promoter activity as compared to the full-length DEC1 promoter reporter construct. However, deletion of the putative TAL1 binding site (-589 region) significantly reduced DEC1 promoter activity. These results suggested that the element which was necessary for high DEC1 promoter activity was likely to reside in the –589 site region. To further determine whether TAL1 was directly associated with DEC1 promoters, Chromatin immunoprecipitation (ChIP) assays were performed. As a negative control, PCR was conducted to amplify the 2000 bp upstream region of the putative TAL1 binding site, which was not expected to associate with DEC1. There was a direct association of TAL1 with putative binding site 3 of DEC1 (Fig. 7g). While there were no interactions of TAL1 with the control region or putative binding site 1.

### TAL1 feedback promoted MIR17HG expression by binding to MIR17HG promoters

We identified putative TAL1 binding sites within the promoter region of MIR17HG using bioinformatic databases (JASPAR CORE). As shown in Fig. 7j, MIR17HG expression was increased when TAL1 was introduced and downregulated in the sh-TAL1 group. Luciferase assays were conducted to assess whether TAL1 might be binding to the MIR17HG promoter. The putative TAL1 binding sites at the -1670 and -785 position in MIR17HG were confirmed. As shown in Additional file 4: Figure S4b, deletion of the -785 site region significantly reduced MIR17HG promoter activity. Furthermore, ChIP assays showed an interaction between TAL1 and the MIR17HG putative binding sites, but no interaction with the control region (Fig. 7k).

### Knockdown of FXR1 and MIR17HG in combination suppressed tumor growth and induced higher survival period in nude mice

**Fig. 8:**
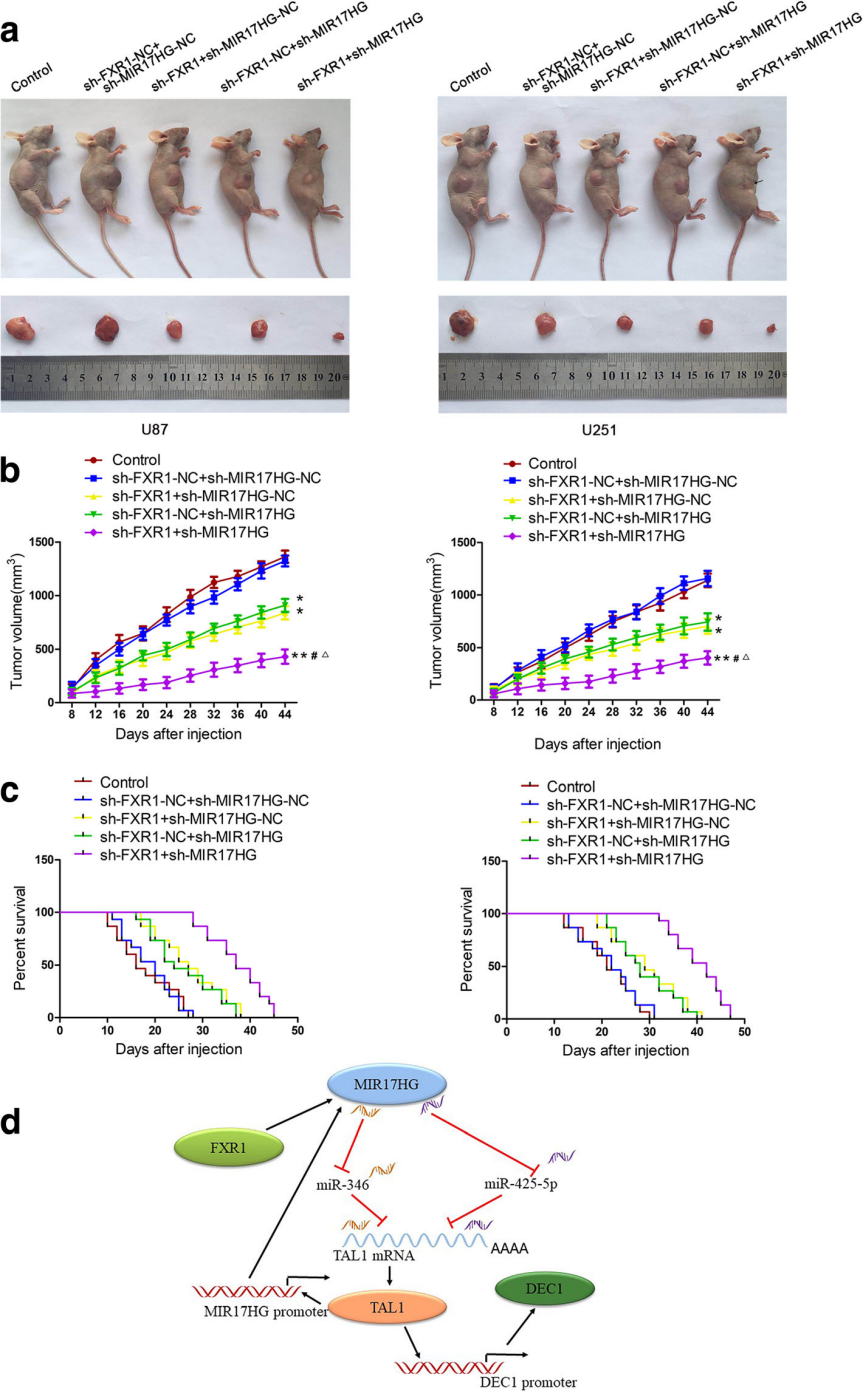
Tumor xenograft studies. **a** The stable expressing cells were used for the in vivo study. The nude mice carrying tumors from respective groups are shown. The sample tumors from respective groups are shown. **b** Tumor volume was calculated every 4 days after injection, and the tumor was excised after 44 days. *P < 0.05, **P < 0.01 versus sh-FXR1-NC + sh-MIR17HG-NC group; ^#^*P* < 0.05 versus sh-FXR1 + sh-MIR17HG-NC group; ^△^*P* < 0.05 versus sh-FXR1-NC + sh-MIR17HG group. Using one-way analysis of variance for statistical analysis. **c** The survival curves of nude mice with xenografts injected into the right striatum (*n* = 15). *P* < 0.05 for sh-FXR1 + sh-MIR17HG-NC or sh-FXR1-NC + sh-MIR17HG versus sh-FXR1-NC + sh-MIR17HG-NC group; *P* < 0.01 for sh-FXR1 + sh-MIR17HG group versus sh-FXR1-NC + sh-MIR17HG-NC group. Using log-rank test for statistical analysis. **d** The schematic cartoon of the mechanism of FXR1 as an oncogene positively regulation of MIR17HG in glioma cells

To further explore the functions of FXR1 and MIR17HG in vivo, stably transfected glioma cells were divided into 5 groups to construct subcutaneous xenografts and orthotopic xenograft models in nude mice. In contrast to sh-FXR1-NC + sh-MIR17HG-NC group, sh-FXR1 + sh-MIR17HG-NC, sh-FXR1-NC + sh-MIR17HG and sh-FXR1 + sh-MIR17HG groups led to smaller tumor volumes. Besides, FXR1 inhibition combined with MIR17HG inhibition produced the smallest tumor volume (Fig. 8a). Further, the survival analysis showed that sh-FXR1, sh-MIR17HG and sh-FXR1 + sh-MIR17HG groups exhibited longer survival period compared with sh-FXR1-NC + sh-MIR17HG-NC group. Moreover, sh-FXR1 + sh-MIR17HG group exhibited the longest survival period (Fig. 8b).

## Discussion

In the present study, we found that expression of FXR1 and MIR17HG were elevated in glioma tissues and cells. Inhibition of FXR1 hindered malignant biological behaviors of glioma cells via destabilizing MIR17HG. On the contrary, miR-346 and miR-425-5p manifested carcinostatic role in glioma cells and suppressed the proliferation, migration and invasion, prompted apoptosis of glioma cells. MIR17HG acted as an endogenous competition RNAs (ceRNAs) sponge preventing miR-346 and miR-425-5p from binding to the 3’UTR of TAL1 mRNA. Mechanistically, knockdown of MIR17HG reduced TAL1 expression through upregulating miR-346/miR-425-5p, which could negatively regulate TAL1 expression by targeting its 3’ UTR. TAL1 exerted oncogenic function by promoting oncogene DEC1 expression. Interestingly, TAL1 upregulated MIR17HG expression by binding to its promoter region. Remarkably, inhibition of FXR1 and MIR17HG in combination largely reduced xenograft tumor growth and prolonged the nude mice survival.

In the present study, we found that FXR1 exhibited high expression levels in glioma tissues and cells. Inhibition of FXR1 hindered malignant biological behaviors of glioma cells. These data suggested that FXR1 functioned as an oncogene in glioma cells. This is in accordance with its tumor promotive role in other cancers. Similar to the results of this study, FXR1 is highly abundant in ovarian and breast cancer, and is associated with poor prognosis. In non-small cell lung cancer, FXR1 is overexpressed in the tissues and cells, silencing of FXR1 exhibited reduced cell growth and elevated cell apoptosis ratio via destabilizing ECT2 mRNA [25]. Moreover, FXR1 expression is upregulated in the squamous cell carcinoma of the head and neck. Degradation of FXR1mediated by Fbxo4 inhibits tumorigenesis in head and neck squamous cell carcinoma, meanwhile, FXR1regulates Fbxo4 expression through inhibition of protein translation in the feedback [26].

Dysregulation of lncRNA is ubiquitous in heterogeneous tumors and were involved in multiple cellular biological processes in tumor cells. In this regard, it is urgent to identify the dysregulated lncRNAs and underlying mechanisms in tumors. In ovarian cancer, LncRNA PTAF expression is upregulated. Overexpressed PTAF induced elevated SNAI2 expression by competitively binding to miR-25, which in turn promoted ovarian cancer cells epithelial-to-mesenchymal transition (EMT) and invasion [27]. In addition, LncRNA HOXD-AS1 could compete with the transcription factor E2F8 to bind with miR-130a, and promoted cell migration and invasion in glioma cells [28]. Our results suggested that MIR17HG expression was positively correlated with the histopathological grade in human glioma tissues and was elevated in U87 and U251 cells. Furthermore, MIR17HG knockdown inhibits the proliferation, migration, and invasion as well as promoting apoptosis in glioma cells, which indicated that MIR17HG may serve as an oncogene facilitated malignant progression of glioma cells.

Post-transcriptional regulatory processes critically affect eukaryotic gene expression programs, which is often controlled by RBPs in conjunction with noncoding RNAs [29]. As critical regulators for RNA metabolism, RBPs can exert a biological effect by regulating target RNAs such as stability [30, 31]. As earlier reported, CPEB4 is highly expressed in glioma tissues. Overexpression of CPEB4 promotes the migration and invasion of glioma cells [32]. In breast cancer, high expression of HuR exerts oncogenic function by increasing the stability of ERBB2 mRNA [33]. Meanwhile, RNA binding protein Lin28A expression was upregulated in osteocarcinoma cells, knockdown of Lin28A inhibited cell proliferation, migration, invasion and promoted cell apoptosis via stabilizing lncRNA MALAT1 [34]. In this study, we found FXR1 expression was positively correlated with MIR17HG expression. FXR1 functions along the same trend with MIR17HG. Further, RIP and RNA pulldown results supported the hypothesis that there was a binding site of FXR1 and MIR17HG. Besides, knockdown of FXR1 decreased MIR17HG expression by reducing MIR17HG’s half-life. Also, knockdown of MIR17HG increased suppression of glioma cell progression induced by inhibition of FXR1. More importantly, knockdown of FXR1 and MIR17HG in combination significantly reduced xenografts tumor growth.

Our data suggested that miR-346 and miR-425-5p expression were declined in glioma tissues and cells. Also, overexpression of miR-346 and miR-425-5p impeded glioma cells malignancy. These findings indicated that miR-346 and miR-425-5p exerted tumor-suppressive function in glioma cells. Earlier reports proved miR-346 suppressed neural stem cells proliferation and promote cells differentiation and apoptosis through directly targeting the 3′UTR of KLF4 and negatively regulates its expression [35]. Moreover, miR-346 expression was reduced in plasma from untreated gastric cancer patients [36]. In addition, miR-425-5p is reduced in osteosarcoma [37]. In acute myeloid leukemia, miR-425-5p was significantly decreased in AML-derived MSC exosomes [38].

Akin to mRNAs, lncRNAs are transcribed into precursor transcripts and are subject to splicing and maturation in the nucleus, as well as cytoplasmic export, editing, transport and decay. In the nucleus and the cytoplasm, lncRNAs are believed to control gene expression by interacting with microRNAs, RBPs, chromatin regulators, transcriptional activators and inhibitors, chromosomal DNA and mRNAs [39, 40]. As previously reported, lncRNA SNHG16 acts as an oncogene by sponging miR-4518 and up-regulating PRMT5 expression in glioma cells [41]. The above findings indicated MIR17HG and miR-346/miR-425-5p had opposite function in glioma cells. miR-346/miR-425-5p expression was negatively correlated with MIR17HG expression. Further, bioinformatics databases and luciferase assay results supported the hypothesis that there was a binding site of MIR17HG and miR-346/miR-425-5p. Besides, knockdown of miR-346/miR-425-5p reversed suppression of glioma cell progression induced by inhibition of MIR17HG. Moreover, compared with inhibition of FXR1 or MIR17HG alone, the inhibition of FXR1 and inhibition of MIR17HG significantly increased the expression of miR-346 and miR-425-5p, further enhanced the suppression of glioma cell malignant progression. These results demonstrated that FXR1 can negatively regulate the expression of miR-346 and miR-425-5p by increasing the stability of MIR17HG, thereby promoting the malignant biological behavior of glioma cells.

Earlier studies confirmed that miRNA are involved in the regulation of cell processes through binding to the 3’UTR of downstream target genes. In esophageal adenocarcinoma cells, reduced levels of miR125a-5p to increase expression of TAP2 through directly targeting TAP2 3’ UTR, which is associated with suppression of anti-tumor immune response and poor outcomes of patients [42]. miR-126 inhibit the cell proliferation and invasion by targeting KRAS mRNA 3’UTR in glioma cells [43].

In the present study, TAL1 was identified as a target of miR-346/miR-425-5p. Our results showed that TAL1 expression was raised in glioma tissues and cells. TAL1 knockdown facilitated inhibition of glioma cell malignant behavior, indicating that TAL1 functions as an oncogene in glioma cells. Consistent with our findings, in human T cell acute lymphoblastic leukemia, TAL1 was overexpressed, and TAL1-mediated up-regulation of miR-223 promotes the malignant phenotype through repression of the FBXW7 tumor suppressor; TAL1 forms a positive interconnected autoregulatory loop with GATA3 and RUNX1 and activates the MYB oncogene to exert oncogenic function [44]*.* In the present studies, Luciferase assays confirmed miR-346 and miR-425-5p targeted TAL1 mRNA 3’ UTR in a sequence-specific manner. In addition, our results showed that overexpression of miR-346/miR-425-5p decreased TAL1 mRNA and protein expression, and inhibited the malignant biological behavior of glioma, while miR-346 and miR-425-5p knockdown showed opposite effects, suggesting that miR-346 and miR-425-5p negatively regulate the expression of TAL1 mRNA and protein at the post-transcriptional level and exert regulatory effects on the biological behavior of glioma cells. Moreover, inhibition of MIR17HG increased the expression of miR-346/miR-425-5p and inhibit the malignant biological behavior of glioma, simultaneous overexpression of miR-346/miR-425-5p increased inhibition of glioma cells by knockdown MIR17HG alone and significantly inhibited TAL1 expression; inhibition of glioma cells caused by MIR17HG (−) was offsetted by miR-346 /miR-425-5p downregulation. The above data revealed that miR-346 /miR-425-5p reduced TAL1 expression by targeting its mRNA 3′-UTR and mediated the tumor-suppressive effect of MIR17HG knockdown. Recent studies have demonstrated the existence of competing endogenous RNAs (ceRNAs). miRNA competitively binds to the transcripts such as lncRNA and mRNA through the miRNA response element (MRE), regulating their expression levels and thus inflecting their functions. Accumulate evidence showed that lncRNAs serve as ceRNAs in affecting the expression and biological functions of miRNA. It has been reported that long non-coding RNA ARNILA functioned as a competing endogenous RNA (ceRNA) sponge preventing miR-204 from binding the 3’UTR of SOX4 mRNA, thereby promoting EMT, invasion and metastasis of triple-negative breast cancer [45]. Similarly, OIP5-AS1 exerted oncogenic function via sponging miR-367-3p and increasing the expression of CEBPA in glioma cells [46]. In addition, the present study found that the binding MRE sequence of MIR17HG and miR-346 (GGCAGAC) is identical to the miR-346 and TAL1 mRNA-3’ UTR binding MRE sequence; the binding MRE sequence of MIR17HG and miR-425-5p (UGUCAU) is identical to that of the miR-346 and TAL1 mRNA-3’ UTR. The above results indicate that MIR17HG acts as ceRNA to bind to miR346 and miR-425-5p, mitigating the negative regulatory effect of miR-346 and miR-425-5p on downstream target gene TAL1, thereby affecting the malignant biological behavior of glioma cells.

TAL1 is identified as a transcription factor and may bind to the specific region of promoters to regulated downstream genes expression. For instance, TAL1 up-regulates *VE-cadherin* gene expression through direct binding to the *VE-cadherin* promoter [47]. Our study demonstrated that DEC1 was up-regulated in glioma tissues and cells. Also, attenuation of DEC1 inhibited the malignant growth of glioma. Furthermore, ChIP assays corroborate our hypothesis that TAL1 directly bind to the DEC1 promoter. Knockdown of TAL1 inhibited DEC1 protein expression, which suggested that TAL1 positively regulate the expression of DEC1 transcriptionally. DEC1 is a member of DEC protein subfamily, which is abundantly expressed in various types of human cancer and is associated with malignant tumor progression. The high expression of DEC1 in gliomas reported here was consistent with the result previously posed, in which showed that DEC1 was overexpressed in glioma tissues and positively correlated with pathological grades [48]. Similarly, DEC1 acts as an anti-apoptotic regulator in gastric cancer cells under hypoxia by promoting Survivin expression [49]. DEC1 was involved in hypoxia-induced epithelial-mesenchymal transition processes via negatively regulating E-cadherin expression in hepatocellular carcinoma HepG2 cells [50]. In breast cancer cells, overexpression of DEC1 promoted the invasiveness of breast cancer through downregulation of claudin-1 [51]. Moreover, overexpression of miR-346/miR-425-5p and TAL1 simultaneously reversed the effect of overexpression of miR-346/miR-425-5p or TAL1 on DEC1 expression, suggesting DEC1 was involved in miR-346(miR-425-5p)/TAL1-mediated regulation of glioma cells.

Interestingly, this study indicated that the transcription factor TAL1 may regulate the expression of the lncRNA MIR17HG, the protein levels of TAL1 had identical changes to the alteration of MIR17HG. Luciferase and ChIP assays also indicated that TAL1 binds to the promoter region of MIR17HG and up-regulates promoter activities. The above findings suggested a positive feedback loop exsit between TAL1 and MIR17HG, knockdown MIR17HG inhibited the expression of transcription factor TAL1 by targeting miR-346 or miR-425-5p, whereas up-regulated TAL1 enhanced MIR17HG expression. Other reports have shown similar mechanisms, which suggested long noncoding RNA SOX2OT upregulated transcription factor SOX3 expression by down-regulating miR-194-5p or miR-122 in glioma cells. SOX3 can also activate SOX2OT promoter to up-regulate its expression [52]. A feedback loop consisting of LINC1410/miR-532-5p/NCF2 was also involved in the progression of gastric cancer cells [53]. Accumulated studies have shown that non-coding RNA-based regulatory networks may play a key role in regulating the malignant behavior of glioma cells.

## Conclusion

we investigated the expression of FXR1, MIR17HG, miR-346, miR-425-5p, TAL1 and DEC1 in glioma cells and elucidated the underlying molecular mechanisms in glioma cells. Our study revealed FXR1exerted oncogenic functions via MIR17HG/miR-346, miR-425-5p/TAL1/DEC1pathway. Our results provide novel theoretical and experimental basis for the pathogenesis of gliomas, and identify novel treatment targets.

## Additional files

Additional file 1: lncRNA microarrays data in U87 and U251 cells. (DOCX 348 kb)

Additional file 2: Transfection efficiency of FXR1, MIR17HG, miR-346, miR-425-5p, TAL1 and DEC1. (DOCX 403 kb)

Additional file 3: TAL1 mRNA expression regulated by miR-346 (miR-425-5p) or MIR17HG and miR-346 (miR-425-5p). (DOCX 613 kb)

Additional file 4: TAL1 on promoter activity of DEC1 and MIR17HG in U87 and U251 cells. (DOCX 203 kb)

## Abbreviations

CCK8: Cell Counting Kit-8
ceRNAs: Endogenous competition RNAs
ChIP: Chromatin immunoprecipitation
FXR: Fragile X-Related
lncRNAs: Long non-coding RNAs
NBTs: Normal brain tissues
NHAs: Normal human astrocytes
qRT-PCR: Quantitative real-time PCR
RBPs: RNA binding proteins
RIP: RNA-binding protein immunoprecipitation
