# Supplementary material for: FXR1 promotes the malignant biological behavior of glioma cells via stabilizing MIR17HG
Source: J Exp Clin Cancer Res. 2019 Jan 28;38:37. doi: 10.1186/s13046-018-0991-0 (PMC6348679; doi:10.1186/s13046-018-0991-0)
Supplement: Supplementary file 1 — lncRNA microarrays data in U87 and U251 cells. (DOCX 348 kb) [file 13046_2018_991_MOESM1_ESM.docx]

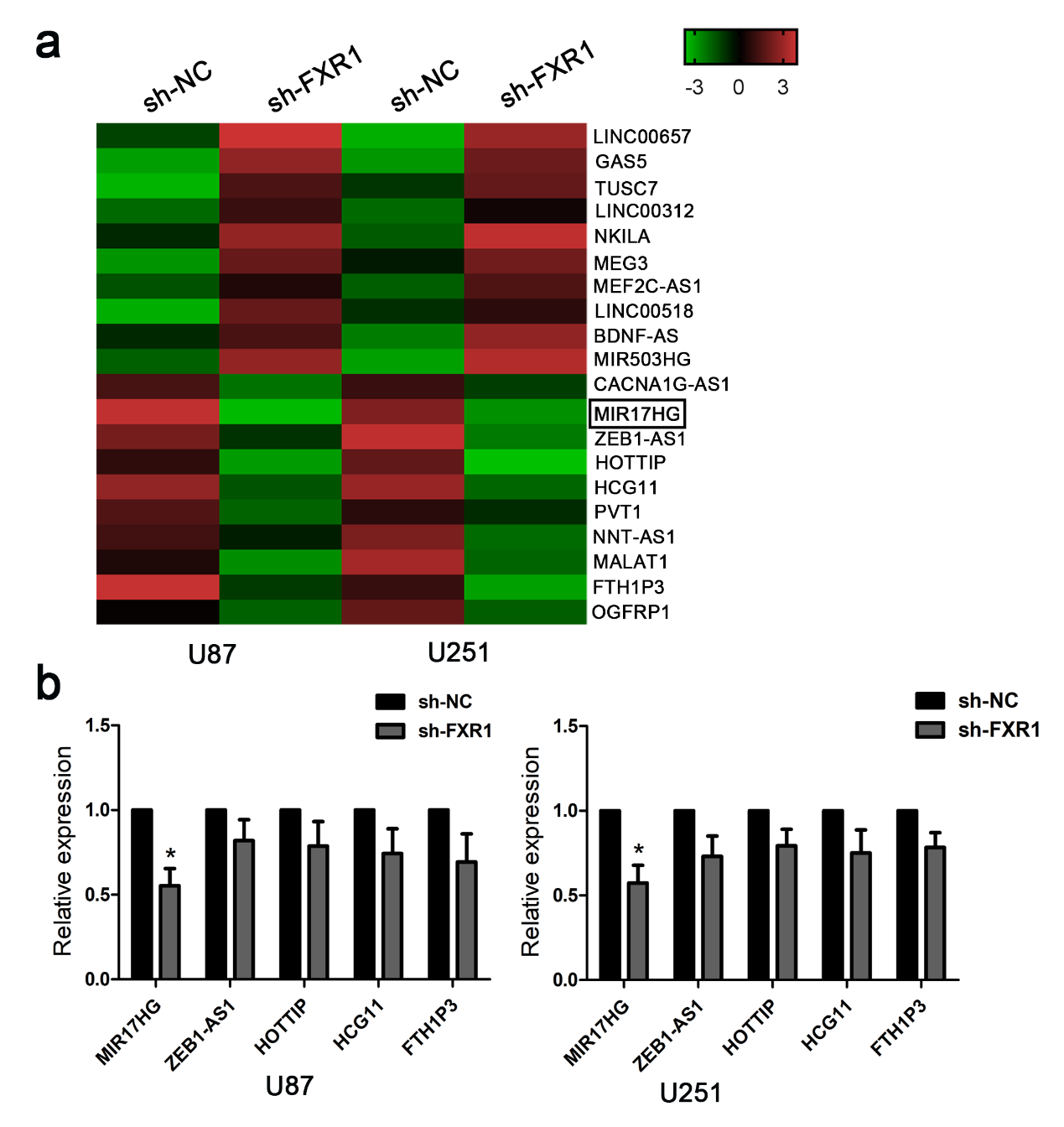


**Supplementary Figure 1. lncRNA microarrays data in U87 and U251 cells.**

**(A)** lncRNA gene expression profiles as obtained from samples in three groups as indicated. **(B)** qRT-PCR was performed to validate the selected molecules. Data are presented as the mean ± SD (n=3 in each group). ******P* < 0.05 versus sh-NC group. Using Student’s t test for statistical analysis.
