# Supplementary material for: FXR1 promotes the malignant biological behavior of glioma cells via stabilizing MIR17HG
Source: J Exp Clin Cancer Res. 2019 Jan 28;38:37. doi: 10.1186/s13046-018-0991-0 (PMC6348679; doi:10.1186/s13046-018-0991-0)
Supplement: Supplementary file 2 — Transfection efficiency of FXR1, MIR17HG, miR-346, miR-425-5p, TAL1 and DEC1. (DOCX 403 kb) [file 13046_2018_991_MOESM2_ESM.docx]

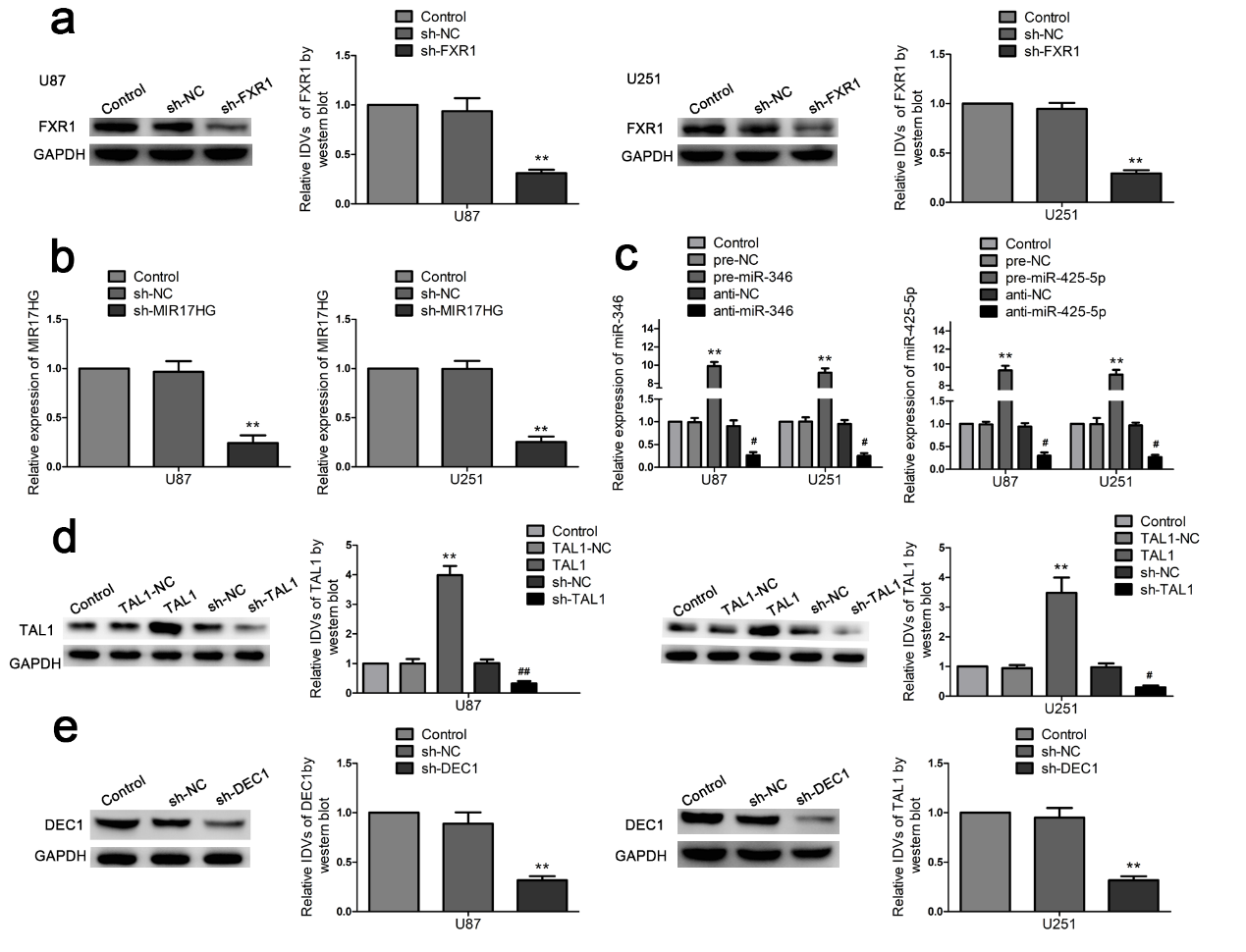


**Supplementary Figure 2.** Transfection efficiency of FXR1, MIR17HG, miR-346，miR-425-5p, TAL1 and DEC1. (a) Western blot was used to investigate the transfection efficiency of FXR1. Data are presented as the mean ± SD (n=3 in each group). *******P* < 0.01 versus sh-NC group (b) qRT-PCR was used to detect the transfection efficiency of MIR17HG. Data represent mean ± SD (n=3 in each group). *******P* < 0.01 versus sh-NC group (c) qRT-PCR was conducted to investigate the transfection efficiency of miR-346 and miR-425-5p. Data are represent mean ± SD (n=3 in each group). *******P* < 0.01 versus pre-NC group, ^#^*P* < 0.05 versus anti-NC group. (d) Western blot was used to investigate the transfection efficiency of TAL1. Data are presented as the mean ± SD (n=3 in each group). *******P* < 0.01 versus TAL1-NC group, ^##^*P* < 0.01, ^#^*P* < 0.05 versus sh-NC group. (e) Western blot was used to investigate the transfection efficiency of DEC1. Data are presented as the mean ± SD (n=3 in each group). *******P* < 0.01 versus sh-NC group. Using one-way analysis of variance for statistical analysis.
