# Supplementary material for: FXR1 promotes the malignant biological behavior of glioma cells via stabilizing MIR17HG
Source: J Exp Clin Cancer Res. 2019 Jan 28;38:37. doi: 10.1186/s13046-018-0991-0 (PMC6348679; doi:10.1186/s13046-018-0991-0)
Supplement: Supplementary file 3 — TAL1 mRNA expression regulated by miR-346 (miR-425-5p) or MIR17HG and miR-346 (miR-425-5p). (DOCX 613 kb) [file 13046_2018_991_MOESM3_ESM.docx]

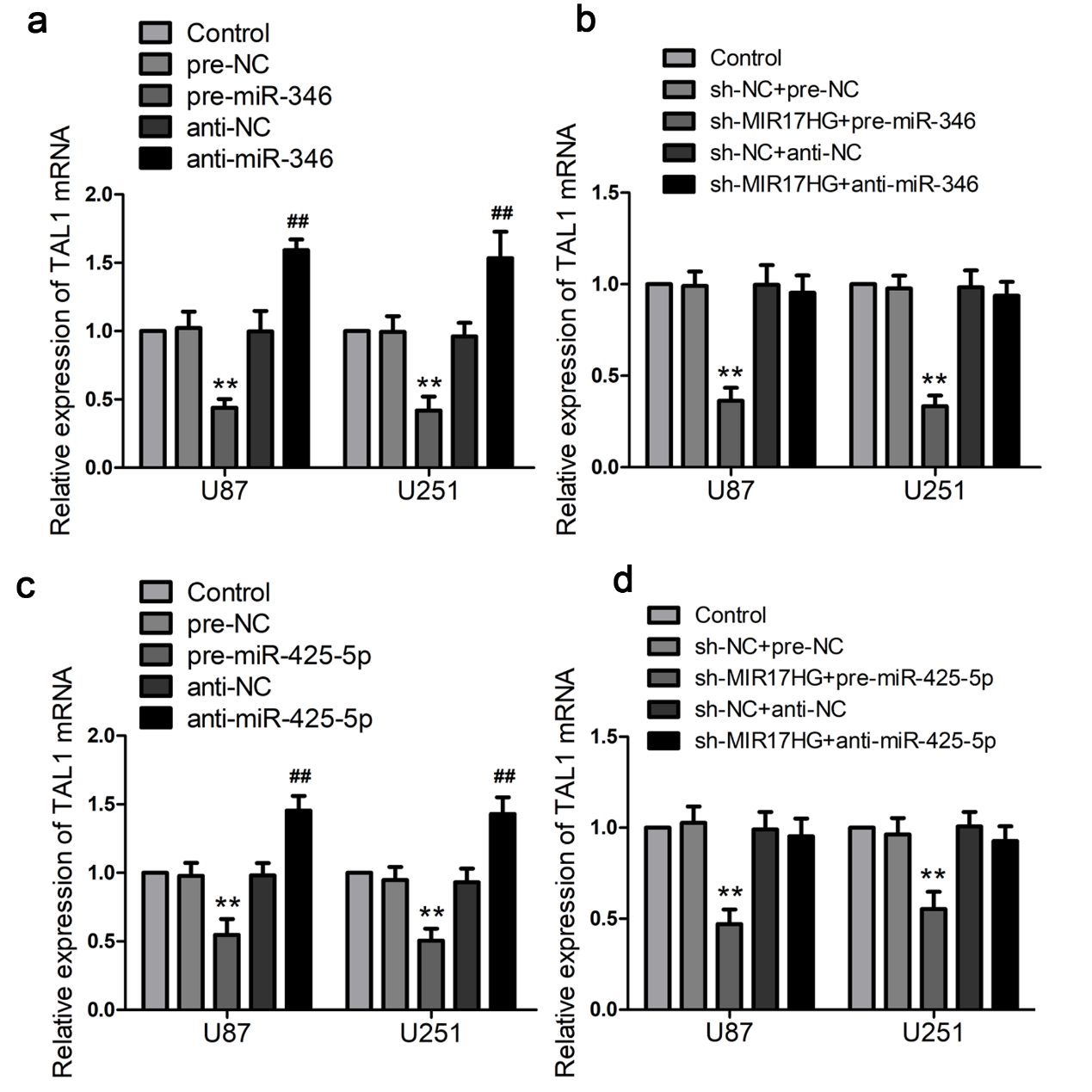


**Supplementary Figure 3.** **TAL1 mRNA expression regulated by miR-346 (miR-425-5p) or MIR17HG and miR-346 (miR-425-5p).**

**(a, c)** qRT-PCR assay was used to detect the TAL1 expression after miR-346 (miR-425-5p) over-expression or knockdown. Data are presented as the mean ± SD (n = 3 in each group). *******P* <0.01 versus pre-NC group; ^##^*p* < 0.01 versus anti-NC group. **(b, d)** qRT-PCR assay was used to detect the TAL1 expression regulated by MIR17HG and miR-346 (miR-425-5p). Data are presented as the mean ± SD (n = 3 in each group). *******P* <0.01 versus sh-NC+pre-NC group. Using one-way analysis of variance for statistical analysis.
