# Supplementary material for: FXR1 promotes the malignant biological behavior of glioma cells via stabilizing MIR17HG
Source: J Exp Clin Cancer Res. 2019 Jan 28;38:37. doi: 10.1186/s13046-018-0991-0 (PMC6348679; doi:10.1186/s13046-018-0991-0)
Supplement: Supplementary file 4 — TAL1 on promoter activity of DEC1 and MIR17HG in U87 and U251 cells. (DOCX 203 kb) [file 13046_2018_991_MOESM4_ESM.docx]

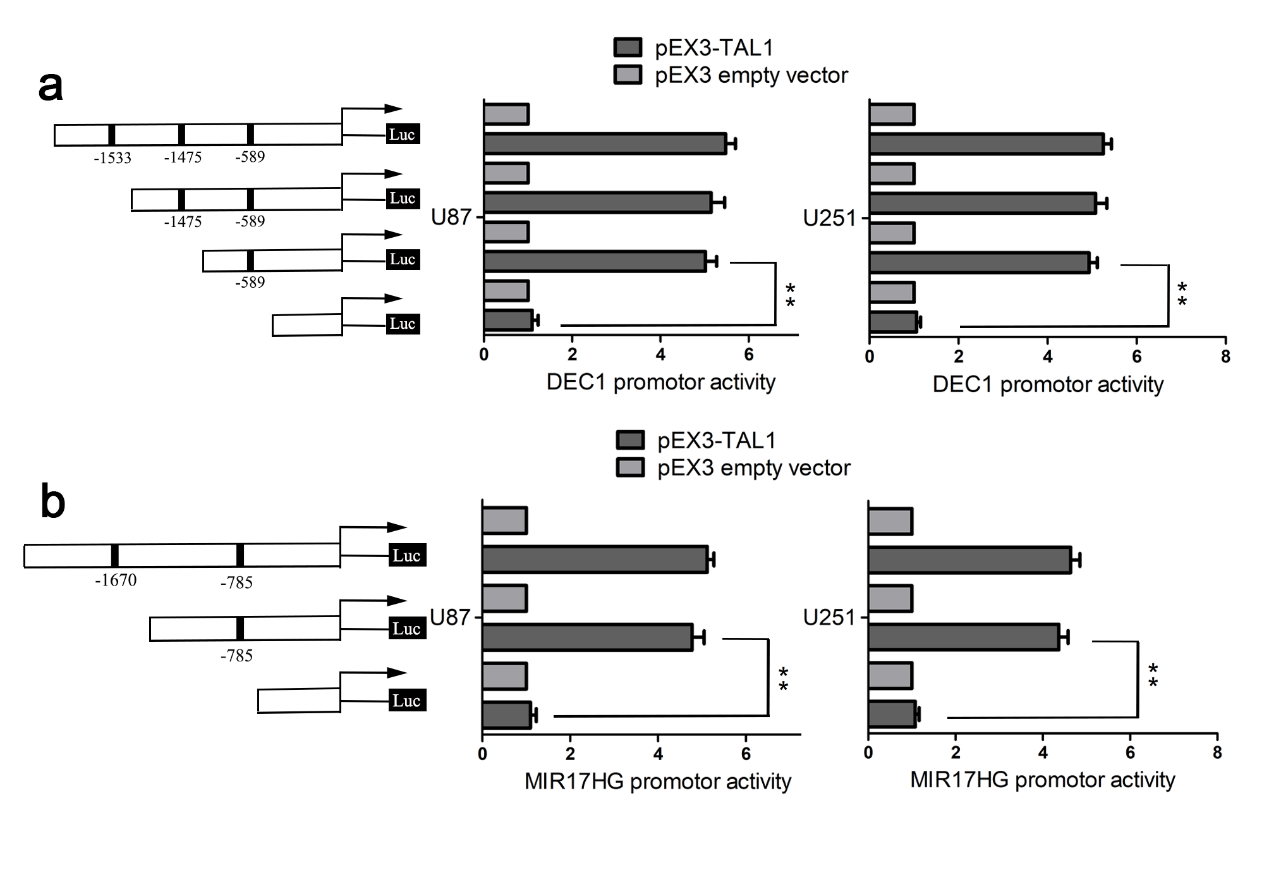


**Supplementary Figure 4：TAL1 on promoter activity of DEC1 and MIR17HG in U87 and U251 cells.** (a, b) Schematic depiction of the different reporter plasmids (a and b) used and the relative luciferase activity are shown. The Y-bar shows the deletion positions on the promoter fragments. X-bar shows the promoter activity of constructed plasmid after the normalization with the co-transfected reference vector (pRL-TK), and expressed as relative to the activity of the pEX3 empty vector, which the activity was set to 1. Data represent mean ± SD (n = 3, each). *******P* < 0.01. Using one-way analysis of variance for statistical analysis.
